# The dynamic RNA modification 5‐methylcytosine and its emerging role as an epitranscriptomic mark

**DOI:** 10.1002/wrna.1510

**Published:** 2018-10-11

**Authors:** Lukas Trixl, Alexandra Lusser

**Affiliations:** ^1^ Division of Molecular Biology Biocenter, Medical University of Innsbruck Innsbruck Austria

**Keywords:** 5‐methylcytosine, epitranscriptomic mark, miRNA, mRNA, N6‐methyladenosine, RNA modification, rRNA, tRNA

## Abstract

It is a well‐known fact that RNA is the target of a plethora of modifications which currently amount to over a hundred. The vast majority of these modifications was observed in the two most abundant classes of RNA, rRNA and tRNA. With the recent advance in mapping technologies, modifications have been discovered also in mRNA and in less abundant non‐coding RNA species. These developments have sparked renewed interest in elucidating the nature and functions of those “epitransciptomic” modifications in RNA. N6‐methyladenosine (m^6^A) is the best understood and most frequent mark of mRNA with demonstrated functions ranging from pre‐mRNA processing, translation, miRNA biogenesis to mRNA decay. By contrast, much less research has been conducted on 5‐methylcytosine (m5C), which was detected in tRNAs and rRNAs and more recently in poly(A)RNAs. In this review, we discuss recent developments in the discovery of m5C RNA methylomes, the functions of m5C as well as the proteins installing, translating and manipulating this modification. Although our knowledge about m5C in RNA transcripts is just beginning to consolidate, it has become clear that cytosine methylation represents a powerful mechanistic strategy to regulate cellular processes on an epitranscriptomic level.

This article is categorized under:

RNA Processing > RNA Editing and Modification
RNA Interactions with Proteins and Other Molecules > Protein–RNA Interactions: Functional Implications
RNA Processing > tRNA Processing
RNA Turnover and Surveillance > Regulation of RNA Stability

## 1 INTRODUCTION

Research on posttranscriptional modification of RNA species has commenced nearly 60 years ago with the description of pseudouridine (Ψ) (Cohn, 1960). Pseudouridine was characterized as a major modification in tRNA and rRNA that is very conserved throughout the prokaryotic and eukaryotic kingdoms (Goodman, Abelson, Landy, Brenner, & Smith, 1968; Zachau, Dütting, & Feldmann, 1966). In later research, the conserved H/ACA snoRNP proteins together with H/ACA snoRNAs were shown to be responsible for installing up to 100 Ψ modifications onto mammalian rRNA (McMahon, Contreras, & Ruggero, 2015). More recently, Ψ residues were also identified in the coding region of mRNAs (Carlile et al., 2014; Lovejoy, Riordan, & Brown, 2014; Schwartz et al., 2014).

The first modifications of mRNA molecules were discovered several decades ago, including the 5′ cap, which contributes among other things to mRNA stability and translation initiation, and the 3′ poly(A) tail that assists the progress of nuclear export, stability as well as translation (Edmonds & Abrams, 1960). Around the same time, the first internal mRNA modifications were described with N6‐methyladenosine (m^6^A) as the most abundant and to date best understood mRNA modification (Dubin & Taylor, 1975; Perry & Kelley, 1975). m^6^A positions were mapped in mRNA upon introduction of an antibody‐mediated detection strategy (Dominissini et al., 2012; Meyer et al., 2012). The discovery of specific “writer”, “reader”, and “eraser” proteins demonstrated that m^6^A is a dynamic modification that affects various aspects of RNA metabolism, including mRNA stability, translation or splicing (reviewed, e.g., in Cao, Li, Yin, & Flavell, 2016; Meyer & Jaffrey, 2017; Peer, Rechavi, & Dominissini, 2017; Schwartz, 2016; Song & Yi, 2017; Zhao, Roundtree, & He, 2016). Based on these findings, the idea was put forward that posttranscriptional modification of RNA might impose information on top of the sequence information contained in the RNA similar to what is known as epigenetic information for DNA and chromatin and prompted the coining of the terms “RNA epigenetics” (He, 2010) and “epitranscriptomics” (Saletore et al., 2012), respectively. Meanwhile, additional modifications have been studied in mRNA, including N1‐methyladenosine (m1A) or 2′‐O‐methylnucleosides, although their functional roles are only beginning to be elucidated (Song & Yi, 2017; Xiong, Li, & Yi, 2018; Zhao et al., 2016).

In this review, we put a spotlight on another modification of RNA that has gained increasing attention in recent years, the methylation of carbon 5 in cytosine (m^5^C). We will discuss methods for its detection, its distribution in different types of RNA, its effects on RNA function and the enzymes responsible for its deposition. Most of our current knowledge about m^5^C in RNA comes from research on the abundant rRNAs and tRNAs. Although we will briefly touch on these findings, in depth discussion of rRNA and tRNA methylation can be found in several excellent recent reviews (e.g., Bohnsack & Sloan, 2018; Sharma & Lafontaine, 2015; Sloan et al., 2017; Sokołowski, Klassen, Bruch, Schaffrath, & Glatt, 2018; Suzuki, Nagao, & Suzuki, 2011; Traube & Carell, 2017). Here, we will concentrate on m^5^C in mRNAs and in less well characterized non‐coding RNA targets.

## 2 METHODS FOR DETECTING M^5^C IN RNA

Methylated cytosine has been discovered first in DNA (Hotchkiss, 1948; Wyatt, 1950) but soon after was also found in RNA (Amos & Korn, 1958). The detection of methylated cytosines in poly(A)RNA was demonstrated in the 1970 for the first time (Desrosiers, Friderici, & Rottman, 1974; Dubin & Taylor, 1975). For these studies, mainly chromatography‐based methods were used to identify methylated nucleotides, including DEAE cellulose chromatography, thin‐layer chromatography and liquid chromatography. Later on, mass spectrometry (MS) proved to be a highly accurate and sensitive method for the identification of RNA modifications especially in combination with liquid chromatography (LC–MS), and the latest techniques in this field allow for detection of RNA modifications in the femto‐ to attomol range. However, the sequence context is usually lost in such analyses because they require enzymatic digestion of RNA to nucleosides (Helm & Motorin, 2017; Kellner, Burhenne, & Helm, 2010). To circumvent this problem approaches have been developed that involve the targeted fragmentation of the RNA by specific enzymes coupled to LC–MS/MS analysis similar to the strategies applied in the proteomics field (Limbach & Paulines, 2017). Yet these approaches still suffer from the lack of suitable bioinformatics resources and tools and relatively low sensitivity that restricts their use to highly abundant RNA classes, such as tRNAs (Wetzel & Limbach, 2013). Top‐down label‐free MS in which RNA is not hydrolyzed was also shown to efficiently identify, localize and quantify methylated nucleobases at a relative level (Glasner, Riml, Micura, & Breuker, 2017). Additionally, studies on human and bacterial ribosomes have revealed high resolution (<3 Å) cryo‐electron microscopy (cryo‐EM) as a powerful tool to simultaneously monitor and localize hundreds of modifications on rRNA (Fischer et al., 2015; Natchiar, Myasnikov, Kratzat, Hazemann, & Klaholz, 2017; Polikanov, Melnikov, Söll, & Steitz, 2015; Shalev‐Benami et al., 2016). However, these techniques are currently not suitable for the analysis of modifications, in particular methylation, in mRNAs, because they require uniform (i.e., one sequence) RNA (top‐down MS) or RNA complexes (cryo‐EM). Thus, current methods used for methylation mapping in mRNA typically are based on RNA sequencing coupled to prior chemical derivatization or enrichment strategies as will be discussed in the following sections.

### 2.1 RNA‐bisulfite‐sequencing

**Figure 1:**
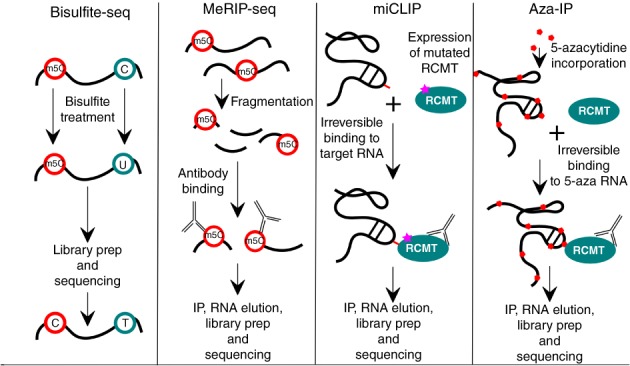
Overview of the most commonly used existing techniques to map m^5^C in RNA

With the development of the bisulfite sequencing technique in 1994, it was possible to study cytosine methylation in DNA in a sequence‐specific manner (Frommer et al. 1992; Clark, Harrison, Paul, & Frommer, 1994). In single stranded DNA, HSO_3_^−^ reacts with cytosine (C) in acidic pH resulting in deamination and formation of uracil‐sulfonate which converts to uracil (U) under basic pH conditions thus causing a C‐to‐U conversion that can be detected by sequencing (Figure 1). In principle, this reaction can also occur on m^5^C, yet it is much slower, which allows for selective distinction between C and m^5^C. In the case of RNA, sodium bisulfite found use in the investigation of amino‐acylation and amino acid acceptance of tRNAs (Chakraburtty, 1975; Sabban & Bhanot, 1982). For RNA methylation studies, however, bisulfite conversion had not been considered as a tool of choice, because of the harsh reaction conditions (denaturation at 95°C, alkali conditions) required, which cause strong degradation of RNA. In 2009, Schäfer et al. reported that by lowering denaturing temperatures and extending incubation times, bisulfite sequencing could also be applied for the detection of m^5^C in RNA which was demonstrated by the identification of m^5^C sites in tRNA and rRNA (Schaefer, Pollex, Hanna, & Lyko, 2009). Unlike any of the other available methods for m^5^C detection (described below), RNA‐bisulfite‐sequencing (RNA‐BS‐seq) allows for the determination of the extent of methylation of a specific C position in RNA. However, one big caveat of RNA‐BS‐seq is its failure to react with Cs in a base‐paired conformation. Considering the low overall m^5^C occurrence in RNA and specifically in mRNA (0.03–0.1% of all Cs; Huber et al., 2015; Legrand et al., 2017), achievement of high C‐to‐U conversion rates is crucial. Thus, efficient denaturation of RNA secondary structures and concomitant best possible preservation of RNA integrity are critical factors determining the robustness of data sets generated by RNA‐BS‐seq (Schaefer, 2015). Recent improvements addressing this issue are the addition of the double‐strand destabilizing agent formamide to the reaction or random fragmentation of the RNA prior to the treatment (Khoddami, Yerra, & Cairns, 2015).

### 2.2 Methylated‐RNA‐immunoprecipitation

Methylated‐RNA‐immunoprecipitation (meRIP‐seq) was originally used to identify the m^6^A methylome in randomly fragmented RNA transcripts (Dominissini et al., 2012; Meyer et al., 2012). This method takes advantage of a highly specific m^6^A antibody for immunoprecipitation and massive parallel sequencing in order to obtain transcriptome wide methylation data. meRIP with an antibody against m^5^C coupled to Illumina sequencing (Figure 1) was used to verify RNA‐BS‐seq results in the archaean *Sulfolobus solfataricus* confirming all of the rRNA and 71% of the mRNA sites detected by RNA‐BS‐seq (Edelheit, Schwartz, Mumbach, Wurtzel, & Sorek, 2013) demonstrating its suitability to detect m^5^C in RNA. However, a drawback to this method is that the antibody reacts preferentially with single stranded nucleic acids (Weber et al., 2005). Thus, RNA secondary structure may obscure potential m^5^C sites from detection.

### 2.3 Aza‐IP and miCLIP

While RNA‐BS‐seq and meRIP‐seq probe m^5^C occurrence without previous knowledge of the nature of the responsible methylating enzyme, aza‐IP and miCLIP were designed to identify the target sites of RNA cytosine methyltransferases (RCMTs). Both techniques take advantage of the catalytic mechanism of RNA methyltransferases which includes a transient covalent linkage of the enzyme to the methylation target cytosine. In aza‐IP, 5‐azacytidine (5‐azaC) is incorporated into RNA by feeding cells with the modified nucleoside. If this occurs at an RCMT target site, it traps the RCMT because the covalent adduct with the target C cannot be resolved. Thus, antibodies against the RCMT (or a tagged version) can be used to immunoprecipitate the protein along with the covalently bound RNA, which is sequenced. Enrichment analysis is then performed to identify methylated RNAs, and sites with increased C‐to‐G transversion signatures (resulting from a ring opening of 5‐azaC during the protocol) correspond to the sites of methylation (Figure 1) (Khoddami & Cairns, 2013). While aza‐IP appears to identify m^5^C sites with high specificity, it may be hampered by limited sensitivity. 5‐azaC is toxic to cells (Flatau, Gonzales, Michalowsky, & Jones, 1984; Jüttermann, Li, & Jaenisch, 1994) necessitating short labeling periods, in which only a small portion of C is replaced by 5‐azaC thereby reducing the probability of being incorporated at the site of a modification. This may be particularly critical for RNAs with low‐expression levels. Along the same lines, some methylation sites may escape detection in enriched sequences since C‐to‐G transversion is not quantitative (Khoddami & Cairns, 2013).

Rather than incorporating a RCMT “suicide” inhibitor, such as 5‐azaC, methylation‐individual nucleotide resolution crosslinking and immmunoprecipitation (miCLIP) is based on the use of a modified RCMT in which the cysteine that is responsible for releasing the RCMT from the RNA substrate is mutated resulting in a covalently linked RNA‐protein complex. Similar to aza‐IP, immunoprecipitation with an antibody against the RCMT is used to enrich bound RNA, which is then subjected to deep sequencing (Figure 1). Because enzyme‐RNA crosslinking leads to termination of reverse transcription at this site, cytosine methylation positions are detected at the +1 site of the sequencing reads rather than by C‐to‐G transversion signatures as in aza‐IP (Hussain et al., 2013). This method does not require incorporation of a modified nucleotide into RNA, but it relies on the overexpression of a mutant RCMT, which may cause changes in methylation patterns. Also, the multistep procedure for detecting the RNAs may lead to decreased data output, which may negatively affect sensitivity.

Taken together, all available methods suffer from different drawbacks caused by a variety of reasons ranging from the biological features of the RNA itself to the experimental procedures and the data analysis approaches (Grozhik & Jaffrey, 2018). Nevertheless, at this point, the complementary use of different methods and carefully designed control experiments should allow for the generation of reliable m^5^C data sets.

## 3 5‐METHYLCYTOSINE IN ABUNDANT RNA SPECIES

Over the years, m^5^C was detected in tRNA (transfer RNA), rRNA (ribosomal RNA), mRNA (messenger RNA), snRNA (small nuclear RNA), miRNA (microRNA), lncRNA (long noncoding RNA) or eRNAs (enhancer RNA) from many species and in all three domains of life. There are differences, however, in the occurrence of m^5^C in specific RNA types in different species. For example, m^5^C appears not to be present in tRNA and mRNA from bacteria, while it has been found in eukaryal and archeal tRNA and mRNA (Amort et al., 2017; Cui et al., 2017; David et al., 2017; Dubin & Taylor, 1975; Edelheit et al., 2013; Fu et al., 2014; Huang et al., 2016; Huber et al., 2015; Motorin, Lyko, & Helm, 2010; Salditt‐Georgieff et al., 1976; Squires et al., 2012; Yang et al., 2017).

### 3.1 tRNA

**Figure 2:**
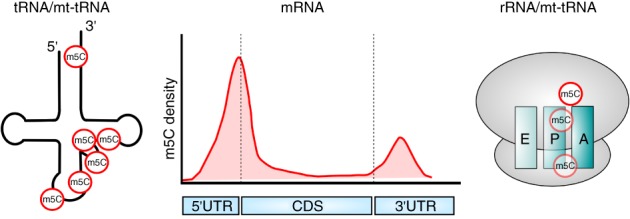
Distribution of identified m^5^C sites in different RNA types. Left, m^5^C positions are marked on a model tRNA. They reside in the acceptor stem (C72), the variable loop region (C47‐C50), the anticodon stem (C38) and the anticodon loop (C34), respectively. Middle, symbolic depiction of the frequency of occurrence of m^5^C along a model mRNA deduced from studies in mouse and humans. Dashed lines mark the translational start and stop codons, respectively. Right, m^5^C positions identified in the 28S/25S rRNA of the large ribosomal subunit and in the 12 rRNA of the small subunit of the mitochondrial ribosome, respectively, are shown. E, P and A sites of the ribosome are symbolized. While the positions of the m^5^C sites in the large ribosomal subunit have been approximately placed according to their position in the ribosomal crystal structure, the m^5^C in the small subunit is shown in an arbitrary location

Most information about m^5^C comes from its study in tRNAs. Methylation occurs most often at cytosines at the junction of the variable loop and the T stem and loop at one, two or three Cs spanning positions 47–50 (Figure 2). It has been proposed that methylation of C48, which forms an unusual “Levitt pair” with nucleoside 15 in the D‐loop to generate the characteristic L‐shape three‐dimensional structure, stabilizes this interaction by increasing the hydrophobicity of the base pair and contributing to base stacking (Väre, Eruysal, Narendran, Sarachan, & Agris, 2017). Another site that is frequently methylated in animals is C38 in the anticodon loop (Figure 2). Methylation of C38 in mouse tRNA^Asp^ was shown to stimulate amino acid charging of the tRNA in vitro and in vivo and to facilitate translation of poly‐Asp containing proteins (Shanmugam et al., 2015). C38 methylation also plays a role in protecting tRNAs from stress‐induced endonuclease‐mediated fragmentation (Schaefer et al., 2010; Tuorto et al., 2012) and in correct translational read‐out of near‐cognate codons (Tuorto et al., 2015). Furthermore, it was shown that in *Schizosaccharomyces pombe*, replacement of guanosine at the wobble position 34 in the anticodon loop by queuosine promotes methylation of C38 providing an example for cross‐talk of different tRNA modifications although the functional significance of this cross‐talk is not yet clear (Ehrenhofer‐Murray, 2017; Jeltsch et al., 2017; Müller et al., 2015). Cytosine methylation was also detected for C34 in tRNA^Leu^_CAA_ and mitochondrial (mt) tRNA^Met^ in mice (Blanco et al., 2014; Trixl et al., 2018) and for mt‐tRNA^Met^ in humans (Haag et al., 2016; Nakano et al., 2016; Van Haute et al., 2016) (Figure 2). C34 methylation of mt‐tRNA^Met^ precedes further modification of this site by oxidation to 5‐formyl‐cytosine (f^5^C), which is important for the decoding of AUA methionine codons during mitochondrial translation (Takemoto et al., 2009). Finally, C72 in human tRNA^Thr^ and tRNA^Cys^ has been shown to be methylated at a late step of tRNA biogenesis, since methylation is dependent on the presence of the posttranscriptionally added CCA sequence at the 3′end (Haag et al., 2015) but so far no specific function has been ascribed to this modification (Figure 2).

### 3.2 rRNA

Ribosomal RNA from all kingdoms of life is subject to cytosine methylation at carbon 5, and the modified positions are fairly conserved throughout evolution (Motorin et al., 2010) (Figure 2). In human and yeast 28S rRNA two methylated cytosines, m^5^C2870 and m^5^C2278, are known (Bourgeois et al., 2015; Motorin et al., 2010; Schaefer et al., 2009; Schosserer et al., 2015; Sharma, Yang, Watzinger, Kötter, & Entian, 2013; Squires et al., 2012), while m^5^C has not been detected in eukaryal 18S RNA (Edelheit et al., 2013). m^5^C2870 resides close to the peptidyltransferase center in the yeast crystal structure of the ribosome, while m^5^C2278 is located at the interface between large and small subunit (Sharma & Lafontaine, 2015). Loss of m^5^C2278 methylation in yeast 25S rRNA resulted in transient changes of rRNA folding upon oxidative stress treatment and it promoted translational read‐through in a reporter construct with a premature stop codon (Schosserer et al., 2015). In the structure of the yeast ribosome, m^5^C2278 along with three other modified bases in the large subunit is contacted by the ribosomal protein eL41, which forms a bridge (eB14) between large and small subunit. This contact was proposed to be important during translation, when eL41 acts as a rotation center for 40S subunit movement (Sharma & Lafontaine, 2015). m^5^C was also detected in mouse mitochondrial 12S but not 16S rRNA (Metodiev et al., 2014) (Figure 2). The function of this modification is currently unknown.

### 3.3 m^5^C IN MRNA

#### 3.3.1 Prevalence of m^5^C sites in mRNA

The existence of m^5^C in eukaryotic mRNA has been known since the 1970s when it was detected in mRNA from HeLa and hamster cells (Dubin & Taylor, 1975; Salditt‐Georgieff et al., 1976), although some earlier studies had failed to detect m^5^C in mammalian cells (Desrosiers et al., 1974; Perry, Kelley, Friderici, & Rottman, 1975). In recent years, improved liquid chromatography‐mass spectrometry (LS‐MS) methods showed that mRNAs indeed possess methylation as well as hydroxymethylation on internal cytosines (Fu et al., 2014; Huang et al., 2016; Huber et al., 2015). Since in LC–MS analysis information regarding the position of m^5^C is lost, the adaptation of bisulfite sequencing for use with RNA unlocked new possibilities to map m^5^C at nucleotide resolution in mRNA (Schaefer et al., 2009). Using this method, in 2012, Preiss and colleagues published the first cytosine methylome for human cells (Squires et al., 2012). They found ~10,000 sites showing >20% methylation and mapping to ~8,500 mRNAs resulting in a rate of 0.43% m^5^C of all sequenced Cs. Recently, the cytosine methylome of mouse embryonic stem cells (ESC) and of the brain was reported (Amort et al., 2017). In this study, ~7,500 m^5^C sites (>20% methylation) mapping to 1,650 mRNAs were detected in ESCs and 2,075 m^5^C sites mapping to 486 mRNAs in the brain. Another recent publication on HeLa cell and mouse cytosine methylomes identified ~3,600 sites within ~2,000 mRNAs in HeLa cells and 2,500–4,400 sites (1,000–1,655 mRNAs) in different mouse tissues (Yang et al., 2017). In the plant *Arabidopsis thaliana*, a few 100 m^5^C sites in mRNA were detected using bisulfite sequencing (David et al., 2017), while another study using meRIP‐seq found 6,045 peaks mapping to 4,465 expressed genes (Cui et al., 2017). meRIP‐seq was also used to examine m^5^C levels in budding yeast and the archaebacterium *Sulfolobus solfataricus* and revealed a single site in yeast and 14 methylated mRNAs in *S. solfataricus* (Edelheit et al., 2013). Finally, a recent study in mouse ESCs applying bisulfite sequencing reported 745 m^5^C sites (Legrand et al., 2017).

These divergent findings by different studies raise two obvious questions: What is the reason for the differences between studies? Does m^5^C in mRNA exist at all? With respect to the first question, it is clear that some of the differences can be attributed to the different methods used. As detailed above, none of the existing techniques to map m^5^C in RNA are without caveats. Secondary structures, sensitivity and specificity issues or data analysis differences, but also different RNA sources (organisms, cell types) may all influence the outcome of m^5^C methylome analyses. Hence, further improvement of the methodology is clearly needed to obtain highly reliable data sets. This will also address the second question of whether mRNA actually contains m^5^C. However, although it is possible that the number of true m^5^C sites in poly(A)RNA in the end will be considerably lower than suggested now, multiple lines of evidence suggest that mRNA is indeed subject to cytosine methylation (Amort et al., 2017; Edelheit et al., 2013; Fu et al., 2014; Huber et al., 2015; Hussain, Aleksic, Blanco, Dietmann, & Frye, 2013; Legrand et al., 2017).

#### 3.3.2 Distribution of m^5^C sites in mRNA

Comparing the findings from different studies available so far revealed that the distribution of m^5^C within mRNA is not random (Figure 2). In HeLa and mouse cells, methylated cytosines were found to be enriched in 5′ and 3′ UTRs and depleted in coding regions (Amort et al., 2017; Squires et al., 2012; Yang et al., 2017). In particular, a pronounced peak of m^5^C was detected in the vicinity of the translational start codon (Amort et al., 2017; Yang et al., 2017) of the cells/tissues examined, while enrichment in the 3′UTR appeared to be rather cell/tissue type specific (Amort et al., 2017). The results from *Arabidopsis* are more conflicting, with one study finding enrichment in the 3′UTR but not in CDS and 5′UTR (David et al., 2017) and the other reporting enrichment in the CDS and depletion in 5′ and 3′UTRs (Cui et al., 2017).

At the level of the individual m^5^C site there is consistent evidence that the modification occurs in a cell/tissue‐type specific manner. In the mouse, the majority of sites detected in different samples was unique to the specific cell/organ (Amort et al., 2017; Yang et al., 2017). Interestingly, the relatively small overlap of sites between ESCs and brain was due to differential methylation rather than differential expression of the target mRNAs at least for the majority of sites detected only in ESCs. In other words, mRNAs that were found to be methylated only in ESCs were expressed but not methylated in the brain. On the other hand, sites detected in the brain samples mapped predominantly to mRNAs that are poorly or not expressed in ESCs (Amort et al., 2017). Likewise, a considerable number of m^5^C sites was specific to a particular mouse tissue although the corresponding mRNA was expressed in all tissues (Yang et al., 2017). In *Arabidopsis,* m^5^C sites that were present in multiple tissues showed differences with respect to the degree of methylation in those tissues (David et al., 2017). Together, these different methylation patterns point towards regulation of cytosine methylation in mRNAs dependent on differentiation type and/or developmental stage.

#### 3.3.3 Function of m^5^C in mRNA

As we are only beginning to uncover the levels and distribution of m^5^C in mRNA, not much is known about the potential functions of this modification. Methylation of cytosine occurs at the Hogsteen edge of the base and therefore does not affect Watson‐Crick base pairing. Because methylation increases the hydrophobicity of the major groove of the RNA, it may have an effect on base stacking (Harcourt, kietrys, & Kool, 2017; Wang & Kool, 1995). The different methylome analyses have attempted to gain information about m^5^C function by performing GO term enrichment analyses of methylated mRNAs or by correlating m^5^C sites with regulatory elements and protein binding sites. In the mouse and in *Arabidopsis*, enrichment of pathways characteristic to the specific cell type analyzed but also of basic cellular and metabolic pathways was detected (Amort et al., 2017; Cui et al., 2017; Yang et al., 2017). By contrast, no such enrichment was found in HeLa cells (Squires et al., 2012). Generally, there was also no correlation between m^5^C occurrence and overall transcript levels (Cui et al., 2017; Yang et al., 2017), own unpublished observation). On the other hand, m^5^C sites have been found to overlap to a certain degree with binding sites of several RNA regulatory proteins, such as Argonaute (Squires et al., 2012) or splicing‐and mRNA decay‐associated factors, such as SRSF3 or UPF (Amort et al., 2017) by correlating m^5^C data with PAR‐CLIP‐data from public databases. However, although enrichment is statistically significant, typically the fraction of overlapping protein binding sites is small and experimental analyses are largely lacking so far. Nevertheless, there are some studies describing specific functions for m^5^C in mRNA metabolism which will be discussed in the following.

#### 3.3.4 m^5^C as a nuclear export regulator

A recent report discovered that the activity of the nuclear export factor ALYREF/THOC4 is strongly affected by the methylation status of its target mRNAs (Yang et al., 2017). mRNAs bound to ALYREF were found to be enriched in m^5^Cs in the vicinity of the translational start codon and in a CG sequence context. Moreover, knock‐down of ALYREF in HeLa cells resulted in increased nuclear retention of m^5^C‐modified mRNA which could be rescued by expression of wild‐type ALYREF but not of a mutant version that was unable to bind m^5^C. In contrast, non‐m^5^C bearing mRNA showed no nuclear export defects upon ALYREF knockdown (Yang et al., 2017). Thus, ALYREF appears to be a bona fide m^5^C “reader” protein with the ability to regulate mRNA fate dependent on its m^5^C status, similar to what has been shown for other proteins in the context of the m^6^A modification (Dominissini & Rechavi, 2017).

#### 3.3.5 m^5^C as a modulator of protein translation

Investigating the molecular mechanisms governing the increase of the cyclin‐dependent kinase inhibitor p27^KIP1^ during replicative senescence, Tang et al. (2015) showed that p27^KIP1^ is subject to cytosine methylation in the 5′UTR and that m^5^C is progressively lost during cell aging. Moreover, it was found that in a cellular reporter gene assay, reporter activity was significantly inhibited upon overexpression of the RNA methyltransferase NSUN2 and that the opposite was true for a NSUN2 knock‐down suggesting that m^5^C introduced by NSUN2 inhibits translation. The latter was also observed in an in vitro translation system arguing against an indirect effect via NSUN2‐mediated tRNA methylation (see later section in this review). Conversely, methylation of specific cytosines in the 3′UTRs of the cell cycle regulators CDK1 and p21, respectively, was shown to promote translation of these mRNAs in vitro and in a reporter gene system in vivo (Li et al., 2017; Xing et al., 2015). Similarly, an m^5^C site within interleukin‐17A mRNA was observed to promote translation of IL‐17A (Wang, Tang, Wang, Wang, & Feng, 2017). However, the precise mechanism by which m^5^C affects translation are presently unknown. In the case of CDK1, NSUN2 knockdown resulted in the association of CDK1 mRNA with a ribosomal fraction containing smaller polysomes, which suggests that m^5^C‐mediated translation regulation might occur at the initiation rather than the elongation level (Xing et al., 2015). Nevertheless, m^5^C may also have the potential to affect ribosomal translation efficiency and to affect decoding potential. Using a bacterial in vitro translation system, Hoernes et al. showed in a systematic analysis of the effect of m^5^C at different codon positions that m^5^C at any position reduced translation efficiency and altered codon specificity when it was inserted at the second position (Hoernes et al., 2016). However, it remains to be shown, if this also holds true for eukaryotic translation.

Together the findings from these studies focusing on m^5^C in specific mRNAs suggest that the position of m^5^C within the mRNA (5′UTR, coding region, 3′UTR) has differential effects on mRNA function.

#### 3.3.6 m5C IN OTHER RNA TYPES

Besides mRNA, tRNA and rRNA, m^5^C has also been detected in other RNA types, specifically in long non‐coding RNAs (lncRNA) and smaller non‐coding RNAs, such as enhancer associated RNAs (eRNAs) or vault RNAs (vtRNAs). For lncRNAs, m^5^C was found near a protein binding domain in human HOTAIR in various cancer cell lines as well as in the functionally important A‐region of the lncRNA XIST. Importantly, m^5^C can interfere with binding of XIST to the chromatin regulatory PRC2 (polycomb repressive complex 2) complex in vitro (Amort et al., 2013). Moreover, the various transcriptome‐wide m^5^C mapping studies also revealed several lncRNAs as methylation targets (Amort et al., 2017; David et al., 2017; Yang et al., 2017). Furthermore, Ribonuclease P RNA component H1 (RPPH1), 5S rRNA, the snoRNA small cajal body specific RNA 2 (SCARNA2), RNY1 and signal recognition particle RNA (7SL RNA) were shown to contain m^5^C by various methods (Hussain, Sajini, et al., 2013; Khoddami & Cairns, 2013; Squires et al., 2012). The functional significance of m^5^C in those RNAs, however, remains elusive so far. m^5^C sites were also identified in vtRNAs using BS‐sequencing, miCLIP and aza‐IP (Hussain, Sajini, et al., 2013; Khoddami & Cairns, 2013; Squires et al., 2012). vtRNAs are RNA components of the vault ribonucleoproteincomplexes the function of which is not yet understood (Kedersha & Rome, 1986). Frye and colleagues showed that the three vtRNAs vtRNA1.1, vtRNA1.2 and vtRNA1.3 contain cytosine methylation sites generated by the methyltransferase NSUN2 and that methylation of vtRNA1.1 affected its processing into smaller fragments (svRNAs). One of these fragments, svRNA4, may have miRNA‐like functions as its increase correlated with a decrease of two potential target mRNAs (Hussain, Sajini, et al., 2013). Another ncRNA target for cytosine methylation that was characterized in some detail are enhancer RNAs (eRNAs) associated with the regulatory regions of several target genes of the transcriptional coregulator peroxisome proliferator‐activated receptor‐gamma coactivator 1 alpha (PGC‐1α) in mouse hepatocytes. PGC‐1α was found to interact with the methyltransferase Nsun7, and depletion of Nsun7 caused a decrease of the presence of m^5^C as well as of the levels of the respective eRNAs suggesting that m^5^C may influence the stability of these eRNAs (Aguilo et al., 2016). As Nsun7 expression in the liver of mice was elevated upon prolonged fasting, concomitant with an increase in overall RNA m^5^C levels, methylation was suggested as a stress‐response mechanism to stabilize eRNA thereby offering a possibility to fine‐tune gene expression under these conditions (Aguilo et al., 2016). Although the number of ncRNA methylation targets that have been studied so far is still small, available data suggest that the m^5^C mark can act as a versatile tool to fine‐tune RNA processing, stability, translation, as well as RNA‐protein interaction.

#### 3.3.7 RNA m^5^C METHYLTRANSFERASES

**Figure 3:**
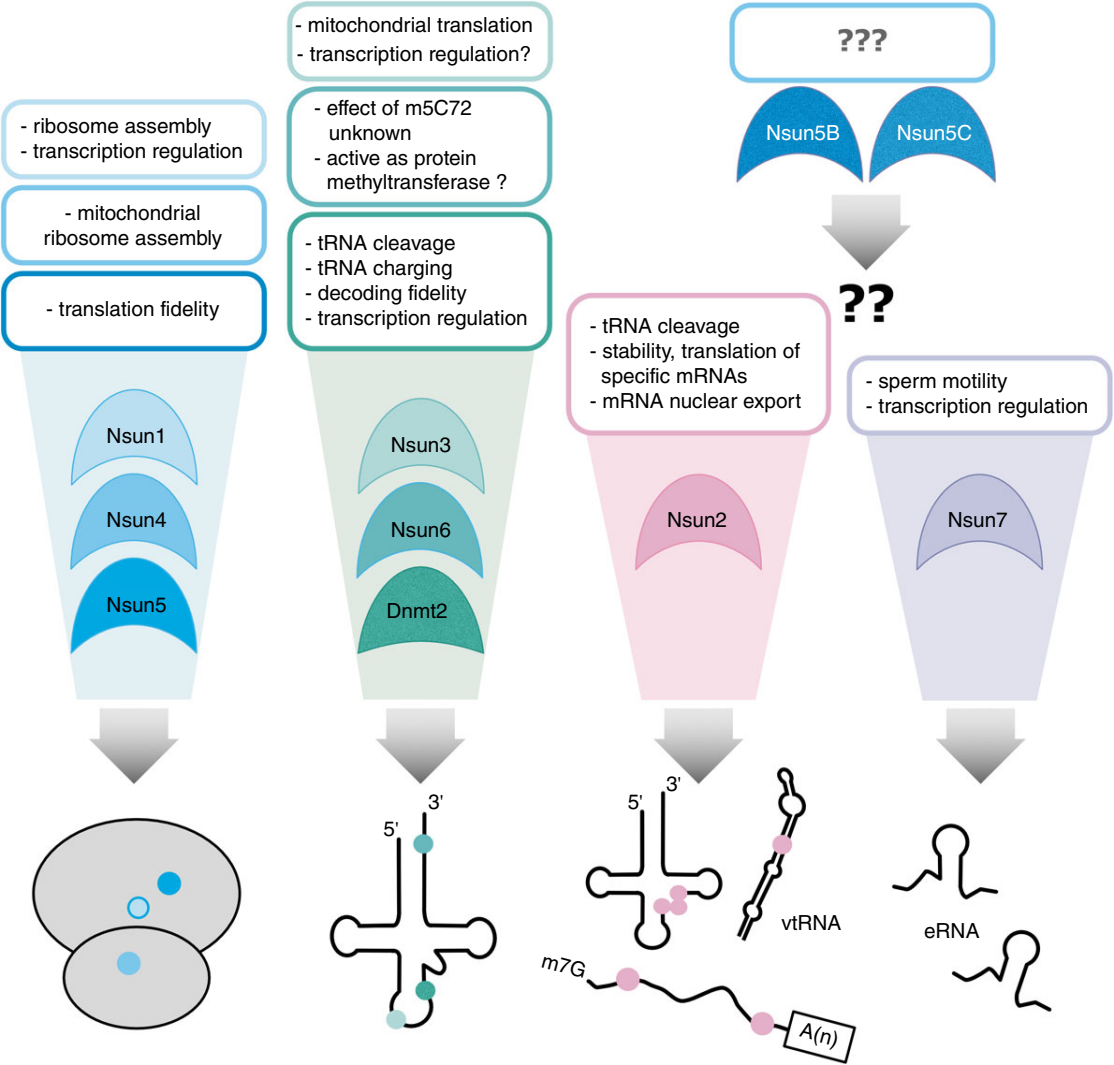
Overview of the different known RCMTs in eukaryotes. The enzymes are grouped according to their substrate preference shown at the bottom part of the figure. The most important consequences of methylation are named in the text at the top

RNA m^5^C methyltransferases belong to the superfamily of Rossman fold‐containing enzymes that use S‐adenosyl‐L‐methionine (SAM) as a methyl group donor. Members of this group of proteins can be found in all domains of life targeting proteins, DNA, RNA, lipids and small molecules for methylation. To date all confirmed m^5^C‐specific RCMTs belong to either the DNMT2 or the NOL1/NOP2/sun (Nsun) subgroups of methyltransferases (Bujnicki, Feder, Ayres, & Redman, 2004; Motorin et al., 2010). In mammals, the Nsun family of enzymes comprises seven genes that include *Nsun1, Nsun2, Nsun3, Nsun4, Nsun5, Nsun6* and *Nsun7*. RCMTs drive the transfer of a methyl group onto cytosine residues of diverse RNA species. All enzymes characterized so far harbor a similar structural core containing the catalytic domain and the SAM binding site. Two conserved cysteine residues that are located in the so‐called motives IV and VI catalyze the methylation reaction by the Nsun family members. The thiol group of the cysteine within motive VI, which is found as a conserved dipeptide motive with threonine (TC), enables the enzyme to covalently bind the target cytosine by attacking the carbon 6 atom of the pyrimidine ring, thus activating the non‐nucleophilic carbon 5 (Liu & Santi, 2000). Extensive protonation leads to a nucleophilic carbon 5 atom that is now susceptible for methylation by electrophilic SAM (Cheng & Roberts, 2001). Beta‐elimination then generates the methylated product and the free enzyme involving the cysteine in motive IV embedded in a dipeptide formation with proline (PC; (King & Redman, 2002). By contrast, DNMT2 lacks the PC motive and, like DNA methyltransferases, uses a one‐cysteine catalytic mechanism (Jeltsch et al., 2017; Jurkowski et al., 2008). Below we will discuss current findings with respect to the substrates and biological roles of eukaryotic RCMTs (Figure 3).

#### 3.3.8 Nsun 1, Nsun 4 and Nsun 5: methylating ribosomal RNA

*Nsun1*: The role of Nsun1 (or Nop2p in S. *cerevisae*) has been investigated already two decades ago, showing a critical involvement in 60S biogenesis in yeast (Sato et al., 1999). Human NSUN1 (also termed NOP2/ nucleolar antigen p120) was found to be strongly overexpressed in multiple human cancers, while normal cell types exhibit low expression levels. Thus, NSUN1 is considered as an effective prognostic marker for cancer development (Jhiang, Yaneva, & Busch, 1990; Uchiyamaet al., 1997; Ueki et al., 1997). Nsun1 localizes predominantly to nucleoli but weaker cytoplasmic staining was also observed in the early developing mouse embryo (Cui et al., 2016). Although long suspected as an RCMT, it was only recently shown to catalyze the transfer of a methyl group onto C2870 of the 25S rRNA in yeast, which strongly affected 60S biogenesis (Sharma et al., 2013). In addition, human NSUN1 was found to be able to complement a yeast *nop2* mutant indicating functional conservation (Bourgeois et al., 2015). Depletion of Nop2p in yeast also compromised the processing of 35S and 27S pre‐rRNAs resulting in lower levels of 25S and 5.8S rRNA. It is not clear yet if this phenotype is related to Nop2p's RCMT function (Hong, Brockenbrough, Wu, & Aris, 1997). In a recent report, human NSUN1 was found to interact with the chromatin regulator bromodomain containing protein 4 (BRD4) and actively elongating RNA polymerase II, and this association was increased in 5‐azacytidine‐resistant versus sensitive leukemia cell lines and in patient derived samples (Cheng et al., 2018) suggesting a role for NSUN1 in transcriptional regulation. It remains unknown at this point if NSUN1 participates in this process by way of its RCMT capacity and what its potential methylation substrates are or if NSUN1 can act as an RNA binding protein in the context of nascent transcription.

*Nsun5*: The enzyme responsible for modifying the second m^5^C position in eukaryotic rRNA is Nsun5 (or Rcm1p in yeast). Rcm1p, which is localized in nucleoli, was identified as the enzyme modifying C2278 in 25S rRNA (Gigova, Duggimpudi, Pollex, Schaefer, & Kos, 2014; Sharma et al., 2013). Loss of *rcm1* resulted in strongly decreased stability of the 60S ribosomal subunit due to impaired association of several ribosomal proteins (Gigova et al., 2014). NSUN5 as well as the *C. elegans* homolog Nsun‐5 were recently found to be downregulated in senescent cells (Schosserer et al., 2015). Interestingly, knock‐down of *Nsun5* in flies, worms and in yeast resulted in extended life span that was dependent on low‐energy nutrition. Moreover, it was observed that reduced Nsun5 levels correlated with decreased translational fidelity and promoted the recruitment of stress‐associated mRNAs to the ribosomal fraction. The physiological downregulation of *Nsun5* during senescence and the concomitantly reduced C2278 rRNA methylation might therefore represent a mechanism to cope with aging‐related effects of stress (Schosserer et al., 2015). In humans, the *NSUN5* gene locus lies in a large chromosomal deletion region that is associated with the multisystemic genetic disorder Williams‐Beuren Syndrome suggesting a contribution of *NSUN5* to the disease phenotype. In this region, two additional copies of *NSUN5* (*NSUN5B, C*) can be found. These genes are transcribed but give rise to shorter open reading frames, and it is currently not clear if the corresponding transcripts are translated (Schubert, 2009).

*Nsun4*: The third rRNA‐specific RCMT is Nsun4. It is imported into mitochondria via an N‐terminal 26 amino acid motif, which is cleaved after its import (Cámara et al., 2011). Nsun4 forms a stoichiometric complex with the mitochondrial regulatory factor MTERF4, which is required for the recruitment of Nsun4 to the large subunit of the mitochondrial ribosome (Cámara et al., 2011). In the absence of MTERF4 (and Nsun4) from the large subunit, mitochondrial ribosome assembly is strongly impaired (Cámara et al., 2011). However, the methylation target of Nsun4 is C911 on the small subunit 12S rRNA and methylation of this site is independent of MTERF4 (Metodiev et al., 2014). Nsun4 inactivation in mice resulted in embryonic lethality, and conditional knock‐out in the heart caused cardiomyopathy with mitochondrial dysfunction underscoring its pivotal role in the regulation of mitochondrial ribosome assembly (Metodiev et al., 2014).

#### 3.3.9 Nsun2: an enzyme with versatile target specificity

Nsun2 was first studied two decades ago in yeast, showing that the disruption of the *Nsun2* gene had neither an effect on ribosome synthesis nor on cell growth, but that cells exhibited increased sensitivity for the antibiotic paromomycin and that Nsun2 localized to the nucleolus in yeast (Wu, Brockenbrough, Paddy, & Aris, 1998). In human cells, NSUN2 was found at different cellular locations in the course of the cell cycle: in G1, it was mostly detected in the nucleolus, during S phase, it was distributed between nucleoli and nucleoplasm, in G2, it localized to the cytoplasm and during M‐phase, it was detected at the centrioles (Frye & Watt, 2006). Site‐specific phosphorylation of Nsun2 at Ser139 by the cell cycle related kinase Aurora B leads to release of Nsun2 from complexes with nucleolar proteins at the onset of mitosis and also significantly reduces its methyltransferase activity (Sakita‐Suto et al., 2007). The first identified Nsun2 methylation substrates were tRNAs. Brzezicha and colleagues showed that NSUN2 was responsible for methylating pre‐tRNA^Leu^ at position C34 in an intron‐dependent manner in HeLa cells (Brzezicha et al., 2006). Further research revealed that Nsun2 methylates most of the transcribed tRNAs at the variable loop region (C47‐C50) (Blanco et al., 2014). In *Nsun2* ^*−/−*^ knock‐out mice, methylation at these positions was lost in tRNA^Gly^, tRNA^Leu^, tRNA^Asp^, and tRNA^Val^, while C38 methylation installed by Dnmt2 was unaffected (Tuorto et al., 2012). Furthermore, *Dnmt2* ^*−/−*^ *Nsun2* ^*−/−*^ double knockout‐mice showed a reduction in total tRNA methylation by more than 90% illustrating that the majority of actively transcribed tRNAs are substrates for Nsun2 and Dnmt2 (Blanco et al., 2014). Accordingly, overall protein synthesis was markedly reduced in *Dnmt2* ^*−/–*^ *Nsun2* ^*−/−*^ mouse embryonic fibroblasts, while translation in single knockout cells was not affected (Tuorto et al., 2012). Besides tRNAs, however, Nsun2 was found to methylate several other ncRNAs (Hussain, Sajini, et al., 2013; Khoddami & Cairns, 2013). For instance, Nsun2 methylates specific vtRNAs, and this modification affects processing of a precursor vtRNA into svRNAs (Hussain, Sajini, et al., 2013). Last but not least, Nsun2 was described in several studies to target mRNAs (Hussain, Sajini, et al., 2013; Li, Li, et al., 2017; Squires et al., 2012; H. Tang et al., 2015; Xing et al., 2015). Recently, it was shown that only overexpression/suppression of NSUN2 but not of any other NSUN enzyme, affected overall m^5^C levels in mRNA from HeLa cells (Yang et al., 2017).

Nsun2 has been implicated in a variety of biological pathways. It was identified as a direct target gene for activation by the transcription factor Myc (Frye & Watt, 2006). Knockdown of Nsun2 resulted in the reduction of Myc‐induced proliferation as well as Myc‐induced terminal differentiation of primary human keratinocytes. Consistent with that, Nsun2 was found to be overexpressed in papillomas and squamous cell carcinomas in the mouse (Frye & Watt, 2006), as well as in a variety of human cancer types (Okamoto et al., 2012). *Dnmt2* ^*−/–*^ *Nsun2* ^*−/−*^ double knockout mice revealed various developmental defects. The mice appeared consistently smaller and lighter than their wild‐type littermates and died before P3. The organization of the cerebral cortex was impaired, and they presented with an immature skeleton with incomplete ossification. Furthermore, altered cellular lipid storage with a strong reduction of brown adipose tissue was observed (Tuorto et al., 2012). Nsun2 was further implicated in testis differentiation (Hussain et al., 2013). Male *Nsun2* ^*−/−*^ mice were sterile due to decreased testes size and a severe reduction in spermatid numbers. Deletion of *Nsun2* leads to the absence of chromatoid bodies, which contain various RNA processing factors, and to abortion of spermatogenesis in the pachytene stage by blocking the progression of prophase I of male meiosis (Hussain, Tuorto, et al., 2013).

In humans, mutations within the *NSUN2* gene have been linked to autosomal‐recessive intellectual disability (Khan et al., 2012). Genotyping of an affected consanguineous Pakistani family revealed a homozygosity‐by‐descent (HBD) locus within region 5p15.32, and a homozygous base substitution was mapped to exon 19 of *NSUN2*, resulting in a glycine to arginine change (G679R). Overall, all individuals showed significant delay in development and speech. Height and weight were below the 5% percentile but muscle tone was increased in all limbs. Although patients had smaller heads, computer tomography of the brain did not show any abnormalities. Expression of mutant NSUN2(G679R) protein in mouse brain prevented its localization to the nucleolus in Purkinje cells of the cerebellum (Khan et al., 2012). A different mutation in the *NSUN2* gene affecting splicing of exons 5–7 was correlated with symptoms of the Dubowitz syndrome, characterized by small stature, intellectual disability, mild microcephaly and a distinct facial appearance. Aberrant splicing resulted in severe reduction of protein levels of the enzyme (Martinez et al., 2012). A recent report provides evidence that the decrease in brain size upon loss of *Nsun2* in mice might derive from the inability to generate sufficient amounts of differentiated neurons during neurogenesis (Flores et al., 2017). Developing *Nsun2* ^*−/−*^ mouse cerebral cortex exhibited a thicker layer and higher numbers of intermediate neuronal progenitors but a decrease in upper‐layer neurons. tRNAs within *Nsun2* ^*−/−*^ brains were found to be hypomethylated and cleaved by angiogenin, which led to an accumulation of 5′ derived tRNA fragments. Indeed, injection of an angiogenin inhibitor into pregnant Nsun2^+/−^ mice rescued the brain development phenotype of the progeny. It was further shown that *Nsun2*‐depleted human neuroepithelial stem cells exhibited migration defects. Thus, it was suggested that Nsun2‐dependent tRNA methylation is required for differentiation and migration of neural progenitor cells during brain development (Flores et al., 2017). Nsun2 has also been implicated in vascular endothelial inflammation and atherosclerosis. Dynamic methylation of the mRNA of intercellular adhesion molecule 1 (ICAM‐1), which is a critical factor for inflammatory and immune responses of the endothelium, upregulated expression of ICAM‐1 at the translational level. Elevated ICAM‐1 levels partly mediated TNF‐α or homocysteine induction of the endothelial inflammatory response, which in turn led to the adhesion of leukocytes to the endothelial cells (Luo, Feng, Xu, Wang, & Wang, 2016).

#### 3.3.10 Dnmt2, Nsun3 and Nsun6: methylating tRNA

*Dnmt2*: In multicellular organisms, Dnmt2 was among the first confirmed RNA m^5^C methyltransferases (Brzezicha et al., 2006; Goll et al., 2006). It was originally thought to act as a DNA methyltransferase, since it shows all sequence and structural characteristics of a DNA methyltransferase, except for a specific nucleic acid binding domain (Dong et al., 2001; Wilkinson, Bartlett, Nurse, & Bird, 1995). However, overall genomic DNA methylation was unaltered in *Dnmt2*‐deficient mice, *Drosophila* and *Arabidopsis,* and Dnmt2 localized predominantly to the cytoplasm (Goll et al., 2006; Okano, Xie, & Li, 1998). Goll et al. showed in in vitro experiments that recombinant Dnmt2 was not able to methylate genomic DNA but instead targeted tRNA^Asp^ for methylation at C38 in the anticodon stem loop (Goll et al., 2006). Later, two additional tRNAs, namely tRNA^Gly^ and tRNA^Val^, were found to be methylated by Dnmt2 at position C38 (Schaefer et al., 2010; Tuorto et al., 2012). Interestingly, all three tRNAs share the same sequence around the methylated C38 (5′ CA‐m^5^C‐GCG 3′), suggesting target recognition by the enzyme in a sequence‐specific manner. *Dnmt2*‐deficient mice, flies and plants were viable and fertile and were morphologically indistinguishable from wild‐type organisms (Goll et al., 2006). Closer inspection of *Dnmt2*‐deficient mice, however, revealed tissue specific differentiation defects reflected in delayed endochondral ossification and hematopoiesis. This phenotype was proposed to be caused by decreased translational fidelity during the decoding of aspartate codons due to the absence of C38 methylation in the responsible tRNA (Tuorto et al., 2015). Another study reported that *Dnmt2*‐deficient mouse cells exhibited increased amounts of uncharged tRNA^Asp^ and impaired synthesis of poly‐asp‐containing proteins (Shanmugamet al., 2015). Thus, *Dnmt2*‐mediated methylation of tRNA^Asp^ C38 appears to be required for proper recognition by its cognate tRNA synthetase, and C38 methylation may be furthermore involved in the decoding of asp codons (Jeltsch et al., 2017). Another phenotype detected in *Dnmt2*‐deficient mice is cardiac hypertrophy. As a potential defect‐mediating mechanism the non‐coding RNA Rn7sk was proposed (Ghanbarian et al., 2016). Rn7sk associates with the transcription elongation regulator P‐Tefb inhibiting its phosphorylation of the C‐terminal domain of RNA polymerase II. In *Dnmt2*‐deficient embryonic stem cells, the association between Rn7sk with P‐Tefb was strongly reduced, and meRIP analysis revealed severely decreased cytosine methylation of Rn7sk isolated from *Dnmt2*‐deficient hearts suggesting that methylation of Rn7sk by Dnmt2 might regulate its interaction with P‐Tefb. Consequently, overactive P‐Tefb might lead to enhanced transcription and cardiac hypertrophy (Ghanbarian et al., 2016). Interestingly, DNMT2 was recently also identified in a complex with P‐TEFb, NSUN3 (see below) and hnRNPK at phospho‐serine 2‐containing active RNA pol II in human leukemia cells. However, the role for DNMT2 in this complex is unknown (Cheng et al., 2018).

While overall *Dnmt2*‐deficient animals show a relatively mild phenotype under standard laboratory conditions, under stress conditions (heat, oxidative, arsenite stress) *Dnmt2*‐deficient flies exhibited shortened life‐span, while dDnmt2 overexpression resulted in increased stress resistance (Lin, Tang, Reddy, & Shen, 2005; Schaefer et al., 2010). These stress‐associated phenotypes of *Dnmt2* mutants might be linked to their translation defects, particularly of poly‐asp containing proteins that might be required for adequate stress response (Shanmugam et al., 2015). Alternatively or in addition, the ability of Dnmt2 to protect tRNA from cleavage by methylating C38 may also play a role in this process. tRNA fragments can impair translation (Gebetsberger & Polacek, 2013; Sobala & Hutvagner, 2013), and in *Drosophila*, they can serve as Dicer 2 (Dcr‐2) substrates and inhibit the action of Dcr‐2 on long double stranded RNAs thus regulating siRNA pathways (Durdevic, Mobin, Hanna, Lyko, & Schaefer, 2013). *Drosophila* mutants of Dnmt2 have also been connected to de‐repression of transposable elements (TE) in the genome (Phalke et al., 2009). A recent report confirmed and expanded these findings and showed that *Nsun2* mutation, too, leads to increased genomic instability. It was suggested that the mechanism by which this occurs is via a disturbance of tRNA metabolism (stability, fragmentation), thus affecting translation of chromatin regulatory proteins involved in the silencing of mobile elements. However, it is also interesting to note that the *Dnmt2*‐deletion phenotype could be rescued by expression of a catalytically inactive *Dnmt2* transgene, which raises the possibility that Dnmt2 might also engage in an RCMT‐independent function in this process (Genenncher et al., 2018). Finally, Dnmt2 was found to be critically required for RNA‐mediated transgenerational inheritance of phenotypic variations. These phenomena are exemplified by the epigenetic modulation of the *Kit* gene, resulting in altered fur coloration of mice, and the modulation of the *Sox9* gene, resulting in an overgrowth phenotype. In *Dnmt2*‐deficient mice the paramutation phenotypes were not transmitted to the next generation. The authors proposed a model in which Dnmt2‐dependent methylation of small paramutation inducing RNAs in the sperm or in the early embryo would protect those RNAs from cleavage thus preserving them to elicit the mutant phenotype (Kiani et al., 2013).

*Nsun3*: One of the latest additions to the group of tRNA‐methylating enzymes is NSUN3. Similar to its closest relative in the Nsun family of RCMTs, it localizes to the mitochondrial matrix in human cells and mouse embryonic stem cells, where it introduces an m^5^C at the “wobble position” C34 of mitochondrial (mt)‐tRNA^Met^ (Haag et al., 2016; Nakano et al., 2016; Trixl et al., 2018; Van Haute et al., 2016). It was shown that the m^5^C modification can be further oxidized by the alpha‐ketoglutarate and Fe(II)‐dependent dioxygenase ALKBH1/ABH1 to generate 5‐formylcytidine (f^5^C) at this position (Haag et al., 2016; Kawarada et al., 2017). This modification is critical for the translation of methionine‐encoding AUA codons in mitochondria (Takemoto et al., 2009). Consistently, mutation of NSUN3 results in reduced mitochondrial protein translation and mitochondrial respiration (Haag et al., 2016; Nakano et al., 2016; Trixl et al., 2018; Van Haute et al., 2016). Catalytic inactivation of Nsun3 in mouse embryonic stem cells further caused impaired differentiation into the neuroectodermal lineage (Trixl et al., 2018). In order to methylate C34, NSUN3 requires an intact anticodon stem loop (Haag et al., 2016) which may explain why two mutations in this region that are associated with mitochondrial disease in humans substantially reduced C34 methylation by NSUN3 in vitro (Nakano et al., 2016). In a recent report, NSUN3 was found to form a complex with hnRNPK, DNMT2 and P‐TEFb at elongating RNA polymerase II sites in leukemia cells implying a role for NSUN3 in transcriptional regulation in the nucleus (Cheng et al., 2018). This is surprising as NSUN3 was so far found to be a mitochondrial protein (Haag et al., 2016; Trixl et al., 2018; Van Haute, Powell, & Minczuk, 2017). Although it was not shown if NSUN3 relocalizes to the nucleus specifically in leukemia cells or if a minor portion of NSUN3 might show nuclear localization in all cell types, it will be interesting to see, whether the proposed nuclear role of NSUN3 involves methylation of nascent mRNA or whether its function is methylation‐independent.

*Nsun6*: This enzyme was shown to reside in the cytoplasm and to partially localize to the Golgi apparatus. Using UV‐crosslinking and analysis of cDNA (CRAC) and aza‐IP Nsun6 was identified as a tRNA‐specific RCMT with specificity for tRNA^Thr^ and tRNA^Cys^ in human cells. It introduces an m^5^C at position 72 in the 3′ acceptor stem. Nsun6 binding to its tRNA substrates requires the presence of a 3′‐CCA sequence in order to carry out methylation, because tRNA^Cys^ and tRNA^Thr^ mutants lacking the 3′‐CCA sequence, were no longer modified at C72 by Nsun6 (Haag et al., 2015). Biochemical and structural analysis of NSUN6 in complex with tRNA^Cys^ revealed further critical determinants of NSUN6 catalytic function. It was found that while the enzyme itself undergoes minor structural changes upon tRNA binding, the conformation of the tRNA at the acceptor stem is markedly altered involving disruption of hydrogen bonds around the target C72 site and nucleotide flipping of the neighboring C71 position to expose C72 for modification. Moreover, the 3′end CCA is bent into a U‐turn for recognition and accommodation by the PUA RNA binding domain of NSUN6 explaining its requirement for catalytic activity. Substrate recognition of NSUN6 is further determined by extensive interactions between the enzyme and the D‐stem region and is dependent on U73, which acts as a discriminator base (Liu, Long, Li, Li, & Wang, 2017; Long et al., 2016). While the role of C72 methylation in tRNA is currently unknown, human NSUN6 has recently been identified to interact with the adaptor protein LLGL2 and the lncRNA MAYA in breast cancer cells. This complex inactivates the kinase Hippo/MST1 by methylation, which in turn allows the MST1 substrate YAP1 to relocate to the nucleus and activate a number of target genes promoting tumor metastasis (Li et al., 2017). Recombinant NSUN6 was shown in in vitro methylation assays to modify MST1, and knockdown in cells reduced the methylation of MST1 (Li, Wang, et al., 2017) raising the intriguing possibility that NSUN6 might be a methyltransferase targeting RNA as well as proteins.

#### 3.3.11 Nsun7: specialized for enhancer RNAs?

According to the Stanford SOURCE database, 50% of all existing NSUN7 protein is testis‐derived. Microarray analysis of mouse testis revealed that Nsun7 expression is the highest in spermatocytes and haploid spermatids (Shima, McLean, McCarrey, & Griswold, 2004). During embryogenesis, however, Nsun7 is broadly expressed in different mouse tissues (Chi & Delgado‐Olguín, 2013). In a forward genetic screen in mice a mutation in exon 7 of Nsun7, which causes a premature stop codon and severe truncation of the protein was linked to impaired male fertility due to reduced motility and aberrant swimming behavior of the sperm (Harris, Marquez, Suarez, & Schimenti, 2007). However, the molecular basis for this defect remains unknown. In a recent report, Nsun7 was found to interact with the transcriptional coactivator PGC‐1α, which contributes to metabolic response of a cell, and to colocalize with PGC‐1α at various target genes. Specifically, knock‐down of Nsun7 reduced enhancer RNA (eRNA) transcripts of PGC‐1α‐controlled genes and this led to a decrease in transcript levels of these genes. Knock‐down of Nsun7 also apparently reduced cytosine methylation in eRNAs suggesting that m^5^C in eRNAs enhances their stability (Aguilo et al., 2016).

## 4 CONCLUSIONS AND PERSPECTIVES

Research on RNA modifications, particularly in mRNA and other lower abundance RNA species, is an emerging and highly dynamic field with new discoveries reported almost on a monthly basis. Yet despite all the exciting new additions to our knowledge, the field is still in its infancy. With respect to m^5^C, this fact is illustrated by seemingly incongruent findings on the nature and distribution of m^5^C in transcriptomes from different sources. Doubtlessly, the refinement and further development of the methodology to map m^5^C on RNA will be an important issue to solve in the future in order to generate high confidence m^5^C methylome data. In this respect, the ongoing improvement of next generation sequencing techniques, such as nanopore sequencing, may hold great potential. Moreover, further development of robust data analysis tools and statistics approaches will be required to deal with large amounts of generated data and to minimize miscalling of m^5^C sites. The elucidation of “writers” and in particular “readers” and “erasers” of the m^5^C epitranscriptomic code is only at its beginning, and discoveries in this area will greatly contribute towards an understanding of the biological functions of m^5^C on RNA. Finally, m^5^C may not be the end of the story. In DNA m^5^C is known to be further oxidized by the TET enzymes to 5‐hydroxymethyl cytosine (hm^5^C), 5‐formylcytosine (f^5^C) and eventually to 5‐carbonylcytosine (ca^5^C). While f^5^C and ca^5^C are mostly considered intermediates on the way to DNA demethylation, hm^5^C has been demonstrated to have regulatory potential on its own. For RNA, a recent report has shown that hm^5^C can be found in mRNAs from *Drosophila*, especially from the fly brain. The *Drosophila* Tet enzyme is responsible for generation of this mark and *Tet*‐mutant flies exhibit impaired brain development and reduced hm^5^C levels (Delatte et al., 2016). Existence of hm^5^C has also been shown for cells of mouse and human origin (Fu et al., 2014; Huber et al., 2015), although these findings could not be confirmed in another study (Legrand et al., 2017). Taken together, it is clear that large areas on the m^5^C epitranscriptomic map are still obscure awaiting discovery in the years to come.

## CONFLICT OF INTEREST

The authors have declared no conflicts of interest for this article.

## RELATED WIREs ARTICLES

mRNA methylation by NSUN2 in cell proliferation

Mapping and significance of the mRNA methylome

RNA methylation in nuclear pre‐mRNA processing

## References

[wrna1510-bib-0001] Aguilo, F. , Li, S. , Balasubramaniyan, N. , Sancho, A. , Benko, S. , Zhang, F. , … Walsh, M. J. (2016). Deposition of 5‐Methylcytosine on enhancer RNAs enables the coactivator function of PGC‐1α. Cell Reports, 14(3), 479–492. 10.1016/j.celrep.2015.12.043 26774474 PMC4731243

[wrna1510-bib-0002] Amort, T. , Rieder, D. , Wille, A. , Khokhlova‐Cubberley, D. , Riml, C. , Trixl, L. , … Lusser, A. (2017). Distinct 5‐methylcytosine profiles in poly(A) RNA from mouse embryonic stem cells and brain. Genome Biology, 18(1), 1 10.1186/s13059-016-1139-1 28077169 PMC5225599

[wrna1510-bib-0003] Amort, T. , Soulière, M. F. , Wille, A. , Jia, X.‐Y. , Fiegl, H. , Wörle, H. , … Lusser, A. (2013). Long non‐coding RNAs as targets for cytosine methylation. RNA Biology, 10(6), 1003–1008. 10.4161/rna.24454 23595112 PMC4111728

[wrna1510-bib-0004] Amos, H. , & Korn, M. (1958). 5‐Methyl cytosine in the RNA of *Escherichia coli* . Biochimica et Biophysica Acta, 29(2), 444–445. 13572373 10.1016/0006-3002(58)90214-2

[wrna1510-bib-0005] Blanco, S. , Dietmann, S. , Flores, J. V. , Hussain, S. , Kutter, C. , Humphreys, P. , … Frye, M. (2014). Aberrant methylation of tRNAs links cellular stress to neuro‐developmental disorders. The EMBO Journal, 33(18), 2020–2039. 10.15252/embj.201489282 25063673 PMC4195770

[wrna1510-bib-0006] Bohnsack, M. T. , & Sloan, K. E. (2018). The mitochondrial epitranscriptome: The roles of RNA modifications in mitochondrial translation and human disease. Cellular and Molecular Life Sciences: CMLS, 75(2), 241–260. 10.1007/s00018-017-2598-6 28752201 PMC5756263

[wrna1510-bib-0007] Bourgeois, G. , Ney, M. , Gaspar, I. , Aigueperse, C. , Schaefer, M. , Kellner, S. , … Motorin, Y. (2015). Eukaryotic rRNA modification by yeast 5‐Methylcytosine‐methyltransferases and human proliferation‐associated antigen p120. PLoS One, 10(7), e0133321. 10.1371/journal.pone.0133321 PMC4510066 26196125

[wrna1510-bib-0008] Brzezicha, B. , Schmidt, M. , Makalowska, I. , Jarmolowski, A. , Pienkowska, J. , & Szweykowska‐Kulinska, Z. (2006). Identification of human tRNA:m5C methyltransferase catalysing intron‐dependent m5C formation in the first position of the anticodon of the pre‐tRNA Leu (CAA). Nucleic Acids Research, 34(20), 6034–6043. 10.1093/nar/gkl765 17071714 PMC1635329

[wrna1510-bib-0009] Bujnicki, J. M. , Feder, M. , Ayres, C. L. , & Redman, K. L. (2004). Sequence‐structure‐function studies of tRNA:m5C methyltransferase Trm4p and its relationship to DNA:m5C and RNA:m5U methyltransferases. Nucleic Acids Research, 32(8), 2453–2463. 10.1093/nar/gkh564 15121902 PMC419452

[wrna1510-bib-0010] Cámara, Y. , Asin‐Cayuela, J. , Park, C. B. , Metodiev, M. D. , Shi, Y. , Ruzzenente, B. , … Larsson, N. G. (2011). MTERF4 regulates translation by targeting the methyltransferase NSUN4 to the mammalian mitochondrial ribosome. Cell Metabolism, 13(5), 527–539. 10.1016/j.cmet.2011.04.002 21531335

[wrna1510-bib-0011] Cao, G. , Li, H.‐B. , Yin, Z. , & Flavell, R. A. (2016). Recent advances in dynamic m6A RNA modification. Open Biology, 6(4), 160003 10.1098/rsob.160003 27249342 PMC4852458

[wrna1510-bib-0012] Carlile, T. M. , Rojas‐Duran, M. F. , Zinshteyn, B. , Shin, H. , Bartoli, K. M. , & Gilbert, W. V. (2014). Pseudouridine profiling reveals regulated mRNA pseudouridylation in yeast and human cells. Nature, 515(7525), 143–146. 10.1038/nature13802 25192136 PMC4224642

[wrna1510-bib-0013] Chakraburtty, K. (1975). Effect of sodium bisulfite modification on the arginine acceptance of *E. coli* tRNA Arg. Nucleic Acids Research, 2(10), 1793–1804. 1103086 10.1093/nar/2.10.1793 PMC343547

[wrna1510-bib-0014] Cheng, J. X. , Chen, L. , Li, Y. , Cloe, A. , Yue, M. , Wei, J. , … Vardiman, J. W. (2018). RNA cytosine methylation and methyltransferases mediate chromatin organization and 5‐azacytidine response and resistance in leukaemia. Nature Communications, 9(1), 1163 10.1038/s41467-018-03513-4 PMC5862959 29563491

[wrna1510-bib-0015] Cheng, X. , & Roberts, R. J. (2001). AdoMet‐dependent methylation, DNA methyltransferases and base flipping. Nucleic Acids Research, 29(18), 3784–3795. 11557810 10.1093/nar/29.18.3784 PMC55914

[wrna1510-bib-0016] Chi, L. , & Delgado‐Olguín, P. (2013). Expression of NOL1/NOP2/sun domain (Nsun) RNA methyltransferase family genes in early mouse embryogenesis. Gene Expression Patterns, 13(8), 319–327. 10.1016/j.gep.2013.06.003 23816522

[wrna1510-bib-0142] Clark, S. J. , Harrison J., Paul, C. L., & Frommer, M. (1994). High sensitivity mapping of methylated cytosines. Nucleic Acids Research, 22(15), 2990–2997. 8065911 10.1093/nar/22.15.2990 PMC310266

[wrna1510-bib-0017] Cohn, W. E. (1960). Pseudouridine, a carbon‐carbon linked ribonucleoside in ribonucleic acids: Isolation, structure, and chemical characteristics. The Journal of Biological Chemistry, 235, 1488–1498. 13811056

[wrna1510-bib-0018] Cui, W. , Pizzollo, J. , Han, Z. , Marcho, C. , Zhang, K. , & Mager, J. (2016). Nop2 is required for mammalian preimplantation development. Molecular Reproduction and Development, 83(2), 124–131. 10.1002/mrd.22600 26632338 PMC4903073

[wrna1510-bib-0019] Cui, X. , Liang, Z. , Shen, L. , Zhang, Q. , Bao, S. , Geng, Y. , … Yu, H. (2017). 5‐Methylcytosine RNA methylation in *Arabidopsis thaliana* . Molecular Plant, 10(11), 1387–1399. 10.1016/j.molp.2017.09.013 28965832

[wrna1510-bib-0020] David, R. , Burgess, A. , Parker, B. , Li, J. , Pulsford, K. , Sibbritt, T. , … Searle, I. R. (2017). Transcriptome‐wide mapping of RNA 5‐Methylcytosine in Arabidopsis mRNAs and non‐coding RNAs. The Plant Cell, 29(3), 445–460. 10.1105/tpc.16.00751 28062751 PMC5385953

[wrna1510-bib-0021] Delatte, B. , Wang, F. , Ngoc, L. V. , Collignon, E. , Bonvin, E. , Deplus, R. , … Fuks, F. (2016). RNA biochemistry. Transcriptome‐wide distribution and function of RNA hydroxymethylcytosine. Science (New York, N.Y.), 351(6270), 282–285. 10.1126/science.aac5253 26816380

[wrna1510-bib-0022] Desrosiers, R. , Friderici, K. , & Rottman, F. (1974). Identification of methylated nucleosides in messenger RNA from Novikoff hepatoma cells. Proceedings of the National Academy of Sciences of the United States of America, 71(10), 3971–3975. 4372599 10.1073/pnas.71.10.3971 PMC434308

[wrna1510-bib-0023] Dominissini, D. , Moshitch‐Moshkovitz, S. , Schwartz, S. , Salmon‐Divon, M. , Ungar, L. , Osenberg, S. , … Rechavi, G. (2012). Topology of the human and mouse m6A RNA methylomes revealed by m6A‐seq. Nature, 485(7397), 201–206. 10.1038/nature11112 22575960

[wrna1510-bib-0024] Dominissini, D. , & Rechavi, G. (2017). 5‐methylcytosine mediates nuclear export of mRNA. Cell Research, 27(6), 717–719. 10.1038/cr.2017.73 28534483 PMC5518879

[wrna1510-bib-0025] Dong, A. , Yoder, J. A. , Zhang, X. , Zhou, L. , Bestor, T. H. , & Cheng, X. (2001). Structure of human DNMT2, an enigmatic DNA methyltransferase homolog that displays denaturant‐resistant binding to DNA. Nucleic Acids Research, 29(2), 439–448. 11139614 10.1093/nar/29.2.439 PMC29660

[wrna1510-bib-0026] Dubin, D. T. , & Taylor, R. H. (1975). The methylation state of poly A‐containing messenger RNA from cultured hamster cells. Nucleic Acids Research, 2(10), 1653–1668. 1187339 10.1093/nar/2.10.1653 PMC343535

[wrna1510-bib-0027] Durdevic, Z. , Mobin, M. B. , Hanna, K. , Lyko, F. , & Schaefer, M. (2013). The RNA methyltransferase Dnmt2 is required for efficient Dicer‐2‐dependent siRNA pathway activity in drosophila. Cell Reports, 4(5), 931–937. 10.1016/j.celrep.2013.07.046 24012760

[wrna1510-bib-0028] Edelheit, S. , Schwartz, S. , Mumbach, M. R. , Wurtzel, O. , & Sorek, R. (2013). Transcriptome‐wide mapping of 5‐methylcytidine RNA modifications in bacteria, archaea, and yeast reveals m5C within archaeal mRNAs. PLoS Genetics, 9(6), e1003602. 10.1371/journal.pgen.1003602 PMC3694839 23825970

[wrna1510-bib-0029] Edmonds, M. , & Abrams, R. (1960). Polynucleotide biosynthesis: Formation of a sequence of adenylate units from adenosine triphosphate by an enzyme from thymus nuclei. The Journal of Biological Chemistry, 235, 1142–1149. 13819354

[wrna1510-bib-0030] Ehrenhofer‐Murray, A. E. (2017). Cross‐talk between Dnmt2‐dependent tRNA methylation and Queuosine modification. Biomolecules, 7(1), 14 10.3390/biom7010014 PMC5372726 28208632

[wrna1510-bib-0031] Fischer, N. , Neumann, P. , Konevega, A. L. , Bock, L. V. , Ficner, R. , Rodnina, M. V. , & Stark, H. (2015). Structure of the *E. coli* ribosome‐EF‐Tu complex at <3 Å resolution by Cs‐corrected cryo‐EM. Nature, 520(7548), 567–570. 10.1038/nature14275 25707802

[wrna1510-bib-0032] Flatau, E. , Gonzales, F. A. , Michalowsky, L. A. , & Jones, P. A. (1984). DNA methylation in 5‐aza‐2′‐deoxycytidine‐resistant variants of C3H 10T1/2 C18 cells. Molecular and Cellular Biology, 4(10), 2098–2102. 6209556 10.1128/mcb.4.10.2098-2102.1984 PMC369027

[wrna1510-bib-0033] Flores, J. V. , Cordero‐Espinoza, L. , Oeztuerk‐Winder, F. , Andersson‐Rolf, A. , Selmi, T. , Blanco, S. , … Frye, M. (2017). Cytosine‐5 RNA methylation regulates neural stem cell differentiation and motility. Stem Cell Reports, 8(1), 112–124. 10.1016/j.stemcr.2016.11.014 28041877 PMC5233436

[wrna1510-bib-0143] Frommer, M. , McDonald, L. E., Millar, D. S., Collis, C. M., Watt, F., Grigg, G. W., … Paul, C. L. (1992). A genomic sequencing protocol that yields a positive display of 5‐methylcytosine residues in individual DNA strands. Proceedings of the National Academy of Sciences of the United States of America, 89(5), 1827–1831. 10.1073/pnas.89.5.1827 1542678 PMC48546

[wrna1510-bib-0034] Frye, M. , & Watt, F. M. (2006). The RNA methyltransferase Misu (NSun2) mediates Myc‐induced proliferation and is upregulated in tumors. Current Biology: CB, 16(10), 971–981. 10.1016/j.cub.2006.04.027 16713953

[wrna1510-bib-0035] Fu, L. , Guerrero, C. R. , Zhong, N. , Amato, N. J. , Liu, Y. , Liu, S. , … Wang, Y. (2014). Tet‐mediated formation of 5‐hydroxymethylcytosine in RNA. Journal of the American Chemical Society, 136(33), 11582–11585. 10.1021/ja505305z 25073028 PMC4140497

[wrna1510-bib-0036] Gebetsberger, J. , & Polacek, N. (2013). Slicing tRNAs to boost functional ncRNA diversity. RNA Biology, 10(12), 1798–1806. 10.4161/rna.27177 24351723 PMC3917982

[wrna1510-bib-0037] Genenncher, B. , Durdevic, Z. , Hanna, K. , Zinkl, D. , Mobin, M. B. , Senturk, N. , … Schaefer, M. (2018). Mutations in Cytosine‐5 tRNA methyltransferases impact Mobile element expression and genome stability at specific DNA repeats. Cell Reports, 22(7), 1861–1874. 10.1016/j.celrep.2018.01.061 29444437

[wrna1510-bib-0038] Ghanbarian, H. , Wagner, N. , Polo, B. , Baudouy, D. , Kiani, J. , Michiels, J.‐F. , … Wagner, K. D. (2016). Dnmt2/Trdmt1 as mediator of RNA polymerase II transcriptional activity in cardiac growth. PLoS One, 11(6), e0156953. 10.1371/journal.pone.0156953 PMC4894585 27270731

[wrna1510-bib-0039] Gigova, A. , Duggimpudi, S. , Pollex, T. , Schaefer, M. , & Kos, M. (2014). A cluster of methylations in the domain IV of 25S rRNA is required for ribosome stability. RNA (New York, NY), 20(10), 1632–1644. 10.1261/rna.043398.113 PMC4174444 25125595

[wrna1510-bib-0040] Glasner, H. , Riml, C. , Micura, R. , & Breuker, K. (2017). Label‐free, direct localization and relative quantitation of the RNA nucleobase methylations m6A, m5C, m3U, and m5U by top‐down mass spectrometry. Nucleic Acids Research, 45(13), 8014–8025. 10.1093/nar/gkx470 28549193 PMC5570050

[wrna1510-bib-0041] Goll, M. G. , Kirpekar, F. , Maggert, K. A. , Yoder, J. A. , Hsieh, C.‐L. , Zhang, X. , … Bestor, T. H. (2006). Methylation of tRNAAsp by the DNA methyltransferase homolog Dnmt2. Science (New York, N.Y.), 311(5759), 395–398. 10.1126/science.1120976 16424344

[wrna1510-bib-0042] Goodman, H. M. , Abelson, J. , Landy, A. , Brenner, S. , & Smith, J. D. (1968). Amber suppression: A nucleotide change in the anticodon of a tyrosine transfer RNA. Nature, 217(5133), 1019–1024. 5643523 10.1038/2171019a0

[wrna1510-bib-0043] Grozhik, A. V. , & Jaffrey, S. R. (2018). Distinguishing RNA modifications from noise in epitranscriptome maps. Nature Chemical Biology, 14(3), 215–225. 10.1038/nchembio.2546 29443978

[wrna1510-bib-0044] Haag, S. , Sloan, K. E. , Ranjan, N. , Warda, A. S. , Kretschmer, J. , Blessing, C. , … Bohnsack, M. T. (2016). NSUN3 and ABH1 modify the wobble position of mt‐tRNAMet to expand codon recognition in mitochondrial translation. The EMBO Journal, 35(19), 2104–2119. 10.15252/embj.201694885 27497299 PMC5048346

[wrna1510-bib-0045] Haag, S. , Warda, A. S. , Kretschmer, J. , Günnigmann, M. A. , Höbartner, C. , & Bohnsack, M. T. (2015). NSUN6 is a human RNA methyltransferase that catalyzes formation of m5C72 in specific tRNAs. RNA (New York, NY), 21(9), 1532–1543. 10.1261/rna.051524.115 PMC4536315 26160102

[wrna1510-bib-0046] Harcourt, E. M. , kietrys, A. M. , & Kool, E. T. (2017). Chemical and structural effects of base modifications in messenger RNA. Nature, 541(7637), 339–346. 10.1038/nature21351 28102265 PMC5498787

[wrna1510-bib-0047] Harris, T. , Marquez, B. , Suarez, S. , & Schimenti, J. (2007). Sperm motility defects and infertility in male mice with a mutation in Nsun7, a member of the sun domain‐containing family of putative RNA methyltransferases. Biology of Reproduction, 77(2), 376–382. 10.1095/biolreprod.106.058669 17442852

[wrna1510-bib-0048] He, C. (2010). Grand challenge commentary: RNA epigenetics? Nature Chemical Biology, 6(12), 863–865. 10.1038/nchembio.482 21079590

[wrna1510-bib-0049] Helm, M. , & Motorin, Y. (2017). Detecting RNA modifications in the epitranscriptome: Predict and validate. Nature Reviews Genetics, 18(5), 275–291. 10.1038/nrg.2016.169 28216634

[wrna1510-bib-0050] Hoernes, T. P. , Clementi, N. , Faserl, K. , Glasner, H. , Breuker, K. , Lindner, H. , … Erlacher, M. D. (2016). Nucleotide modifications within bacterial messenger RNAs regulate their translation and are able to rewire the genetic code. Nucleic Acids Research, 44(2), 852–862. 10.1093/nar/gkv1182 26578598 PMC4737146

[wrna1510-bib-0051] Hong, B. , Brockenbrough, J. S. , Wu, P. , & Aris, J. P. (1997). Nop2p is required for pre‐rRNA processing and 60S ribosome subunit synthesis in yeast. Molecular and Cellular Biology, 17(1), 378–388. 8972218 10.1128/mcb.17.1.378 PMC231762

[wrna1510-bib-0052] Hotchkiss, R. D. (1948). The quantitative separation of purines, pyrimidines, and nucleosides by paper chromatography. The Journal of Biological Chemistry, 175(1), 315–332. 18873306

[wrna1510-bib-0053] Huang, W. , Lan, M.‐D. , Qi, C.‐B. , Zheng, S.‐J. , Wei, S.‐Z. , Yuan, B.‐F. , & Feng, Y.‐Q. (2016). Formation and determination of the oxidation products of 5‐methylcytosine in RNA. Chemical Science, 7(8), 5495–5502. 10.1039/C6SC01589A 30034689 PMC6021781

[wrna1510-bib-0054] Huber, S. M. , van Delft, P. , Mendil, L. , Bachman, M. , Smollett, K. , Werner, F. , … Balasubramanian, S. (2015). Formation and abundance of 5‐hydroxymethylcytosine in RNA. Chembiochem, 16(5), 752–755. 10.1002/cbic.201500013 25676849 PMC4471624

[wrna1510-bib-0055] Hussain, S. , Aleksic, J. , Blanco, S. , Dietmann, S. , & Frye, M. (2013). Characterizing 5‐methylcytosine in the mammalian epitranscriptome. Genome Biology, 14(11), 215–210. 10.1186/gb4143 24286375 PMC4053770

[wrna1510-bib-0056] Hussain, S. , Sajini, A. A. , Blanco, S. , Dietmann, S. , Lombard, P. , Sugimoto, Y. , … Frye, M. (2013). NSun2‐mediated cytosine‐5 methylation of vault noncoding RNA determines its processing into regulatory small RNAs. Cell Reports, 4(2), 255–261. 10.1016/j.celrep.2013.06.029 23871666 PMC3730056

[wrna1510-bib-0057] Hussain, S. , Tuorto, F. , Menon, S. , Blanco, S. , Cox, C. , Flores, J. V. , … Frye, M. (2013). The mouse cytosine‐5 RNA methyltransferase NSun2 is a component of the chromatoid body and required for testis differentiation. Molecular and Cellular Biology, 33(8), 1561–1570. 10.1128/MCB.01523-12 23401851 PMC3624257

[wrna1510-bib-0058] Jeltsch, A. , Ehrenhofer‐Murray, A. , Jurkowski, T. P. , Lyko, F. , Reuter, G. , Ankri, S. , … Helm, M. (2017). Mechanism and biological role of Dnmt2 in nucleic acid methylation. RNA Biology, 14(9), 1108–1123. 10.1080/15476286.2016.1191737 27232191 PMC5699548

[wrna1510-bib-0059] Jhiang, S. M. , Yaneva, M. , & Busch, H. (1990). Expression of human proliferation‐associated nucleolar antigen p120. Cell Growth & Differentiation, 1(7), 319–324. 2278884

[wrna1510-bib-0060] Jurkowski, T. P. , Meusburger, M. , Phalke, S. , Helm, M. , Nellen, W. , Reuter, G. , & Jeltsch, A. (2008). Human DNMT2 methylates tRNA(Asp) molecules using a DNA methyltransferase‐like catalytic mechanism. RNA (New York, NY), 14(8), 1663–1670. 10.1261/rna.970408 PMC2491481 18567810

[wrna1510-bib-0061] Jüttermann, R. , Li, E. , & Jaenisch, R. (1994). Toxicity of 5‐aza‐2′‐deoxycytidine to mammalian cells is mediated primarily by covalent trapping of DNA methyltransferase rather than DNA demethylation. Proceedings of the National Academy of Sciences of the United States of America, 91(25), 11797–11801. 7527544 10.1073/pnas.91.25.11797 PMC45322

[wrna1510-bib-0062] Kawarada, L. , Suzuki, T. , Ohira, T. , Hirata, S. , Miyauchi, K. , & Suzuki, T. (2017). ALKBH1 is an RNA dioxygenase responsible for cytoplasmic and mitochondrial tRNA modifications. Nucleic Acids Research, 45(12), 7401–7415. 10.1093/nar/gkx354 28472312 PMC5499545

[wrna1510-bib-0063] Kedersha, N. L. , & Rome, L. H. (1986). Isolation and characterization of a novel ribonucleoprotein particle: Large structures contain a single species of small RNA. The Journal of Cell Biology, 103(3), 699–709. 2943744 10.1083/jcb.103.3.699 PMC2114306

[wrna1510-bib-0064] Kellner, S. , Burhenne, J. , & Helm, M. (2010). Detection of RNA modifications. RNA Biology, 7(2), 237–247. 20224293 10.4161/rna.7.2.11468

[wrna1510-bib-0065] Khan, M. A. , Rafiq, M. A. , Noor, A. , Hussain, S. , Flores, J. V. , Rupp, V. , … Vincent, J. B. (2012). Mutation in NSUN2, which encodes an RNA methyltransferase, causes autosomal‐recessive intellectual disability. American Journal of Human Genetics, 90(5), 856–863. 10.1016/j.ajhg.2012.03.023 22541562 PMC3376419

[wrna1510-bib-0066] Khoddami, V. , & Cairns, B. R. (2013). Identification of direct targets and modified bases of RNA cytosine methyltransferases. Nature Biotechnology, 31(5), 458–464. 10.1038/nbt.2566 PMC3791587 23604283

[wrna1510-bib-0067] Khoddami, V. , Yerra, A. , & Cairns, B. R. (2015). Experimental approaches for target profiling of RNA cytosine methyltransferases. Methods in Enzymology, 560, 273–296. 10.1016/bs.mie.2015.03.008 26253975

[wrna1510-bib-0068] Kiani, J. , Grandjean, V. , Liebers, R. , Tuorto, F. , Ghanbarian, H. , Lyko, F. , … Rassoulzadegan, M. (2013). RNA‐mediated epigenetic heredity requires the cytosine methyltransferase Dnmt2. PLoS Genetics, 9(5), e1003498. 10.1371/journal.pgen.1003498 PMC3662642 23717211

[wrna1510-bib-0069] King, M. Y. , & Redman, K. L. (2002). RNA methyltransferases utilize two cysteine residues in the formation of 5‐methylcytosine. Biochemistry, 41(37), 11218–11225. 12220187 10.1021/bi026055q

[wrna1510-bib-0070] Legrand, C. , Tuorto, F. , Hartmann, M. , Liebers, R. , Jacob, D. , Helm, M. , & Lyko, F. (2017). Statistically robust methylation calling for whole‐transcriptome bisulfite sequencing reveals distinct methylation patterns for mouse RNAs. Genome Research, 27(9), 1589–1596. 10.1101/gr.210666.116 28684555 PMC5580717

[wrna1510-bib-0071] Li, C. , Wang, S. , Xing, Z. , Lin, A. , Liang, K. , Song, J. , … Yang, L. (2017). A ROR1‐HER3‐lncRNA signalling axis modulates the Hippo‐YAP pathway to regulate bone metastasis. Nature Cell Biology, 19(2), 106–119. 10.1038/ncb3464 28114269 PMC5336186

[wrna1510-bib-0072] Li, Q. , Li, X. , Tang, H. , Jiang, B. , Dou, Y. , Gorospe, M. , & Wang, W. (2017). NSUN2‐mediated m5C methylation and METTL3/METTL14‐mediated m6A methylation cooperatively enhance p21 translation. Journal of Cellular Biochemistry, 118(9), 2587–2598. 10.1002/jcb.25957 28247949 PMC5509477

[wrna1510-bib-0073] Limbach, P. A. , & Paulines, M. J. (2017). Going global: The new era of mapping modifications in RNA. Wiley Interdisciplinary Reviews RNA, 8(1), e1367. 10.1002/wrna.1367 PMC5133204 27251302

[wrna1510-bib-0074] Lin, M.‐J. , Tang, L.‐Y. , Reddy, M. N. , & Shen, C.‐K. J. (2005). DNA methyltransferase gene dDnmt2 and longevity of Drosophila. The Journal of Biological Chemistry, 280(2), 861–864. 10.1074/jbc.C400477200 15533947

[wrna1510-bib-0075] Liu, R.‐J. , Long, T. , Li, J. , Li, H. , & Wang, E.‐D. (2017). Structural basis for substrate binding and catalytic mechanism of a human RNA:m5C methyltransferase NSun6. Nucleic Acids Research, 45(11), 6684–6697. 10.1093/nar/gkx473 28531330 PMC5499824

[wrna1510-bib-0076] Liu, Y. , & Santi, D. V. (2000). m5C RNA and m5C DNA methyl transferases use different cysteine residues as catalysts. Proceedings of the National Academy of Sciences of the United States of America, 97(15), 8263–8265. 10899996 10.1073/pnas.97.15.8263 PMC26935

[wrna1510-bib-0077] Long, T. , Li, J. , Li, H. , Zhou, M. , Zhou, X.‐L. , Liu, R.‐J. , & Wang, E.‐D. (2016). Sequence‐specific and shape‐selective RNA recognition by the human RNA 5‐Methylcytosine methyltransferase NSun6. The Journal of Biological Chemistry, 291(46), 24293–24303. 10.1074/jbc.M116.742569 27703015 PMC5104949

[wrna1510-bib-0078] Lovejoy, A. F. , Riordan, D. P. , & Brown, P. O. (2014). Transcriptome‐wide mapping of pseudouridines: Pseudouridine synthases modify specific mRNAs in *Scerevisiae* . PLoS ONE, 9(10), e110799. 10.1371/journal.pone.0110799 PMC4212993 25353621

[wrna1510-bib-0079] Luo, Y. , Feng, J. , Xu, Q. , Wang, W. , & Wang, X. (2016). NSun2 deficiency protects endothelium from inflammation via mRNA methylation of ICAM‐1. Circulation Research, 118(6), 944–956. 10.1161/CIRCRESAHA.115.307674 26838785

[wrna1510-bib-0080] Martinez, F. J. , Lee, J. H. , Lee, J. E. , Blanco, S. , Nickerson, E. , Gabriel, S. , … Gleeson, J. G. (2012). Whole exome sequencing identifies a splicing mutation in NSUN2 as a cause of a Dubowitz‐like syndrome. Journal of Medical Genetics, 49(6), 380–385. 10.1136/jmedgenet-2011-100686 22577224 PMC4771841

[wrna1510-bib-0081] McMahon, M. , Contreras, A. , & Ruggero, D. (2015). Small RNAs with big implications: New insights into H/ACA snoRNA function and their role in human disease. Wiley Interdisciplinary Reviews RNA, 6(2), 173–189. 10.1002/wrna.1266 25363811 PMC4390053

[wrna1510-bib-0082] Metodiev, M. D. , Spåhr, H. , Loguercio Polosa, P. , Meharg, C. , Becker, C. , Altmueller, J. , … Ruzzenente, B. (2014). NSUN4 is a dual function mitochondrial protein required for both methylation of 12S rRNA and coordination of mitoribosomal assembly. PLoS Genetics, 10(2), e1004110. 10.1371/journal.pgen.1004110 PMC3916286 24516400

[wrna1510-bib-0083] Meyer, K. D. , & Jaffrey, S. R. (2017). Rethinking m6A Readers, Writers, and Erasers. Annual Review of Cell and Developmental Biology, 33(1), 319–342. 10.1146/annurev-cellbio-100616-060758 PMC5963928 28759256

[wrna1510-bib-0084] Meyer, K. D. , Saletore, Y. , Zumbo, P. , Elemento, O. , Mason, C. E. , & Jaffrey, S. R. (2012). Comprehensive analysis of mRNA methylation reveals enrichment in 3' UTRs and near stop codons. Cell, 149(7), 1635–1646. 10.1016/j.cell.2012.05.003 22608085 PMC3383396

[wrna1510-bib-0085] Motorin, Y. , Lyko, F. , & Helm, M. (2010). 5‐methylcytosine in RNA: Detection, enzymatic formation and biological functions. Nucleic Acids Research, 38(5), 1415–1430. 10.1093/nar/gkp1117 20007150 PMC2836557

[wrna1510-bib-0086] Müller, M. , Hartmann, M. , Schuster, I. , Bender, S. , Thüring, K. L. , Helm, M. , … Ehrenhofer‐Murray, A. E. (2015). Dynamic modulation of Dnmt2‐dependent tRNA methylation by the micronutrient queuine. Nucleic Acids Research, 43(22), 10952–10962. 10.1093/nar/gkv980 26424849 PMC4678861

[wrna1510-bib-0087] Nakano, S. , Suzuki, T. , Kawarada, L. , Iwata, H. , Asano, K. , & Suzuki, T. (2016). NSUN3 methylase initiates 5‐formylcytidine biogenesis in human mitochondrial tRNA(met). Nature Chemical Biology, 12(7), 546–551. 10.1038/nchembio.2099 27214402

[wrna1510-bib-0088] Natchiar, S. K. , Myasnikov, A. G. , Kratzat, H. , Hazemann, I. , & Klaholz, B. P. (2017). Visualization of chemical modifications in the human 80S ribosome structure. Nature, 551(7681), 472 10.1038/nature24482 29143818

[wrna1510-bib-0089] Okamoto, M. , Hirata, S. , Sato, S. , Koga, S. , Fujii, M. , Qi, G. , … Tatsuka, M. (2012). Frequent increased gene copy number and high protein expression of tRNA (cytosine‐5‐)‐methyltransferase (NSUN2) in human cancers. DNA and Cell Biology, 31(5), 660–671. 10.1089/dna.2011.1446 22136356

[wrna1510-bib-0090] Okano, M. , Xie, S. , & Li, E. (1998). Dnmt2 is not required for de novo and maintenance methylation of viral DNA in embryonic stem cells. Nucleic Acids Research, 26(11), 2536–2540. 9592134 10.1093/nar/26.11.2536 PMC147598

[wrna1510-bib-0091] Peer, E. , Rechavi, G. , & Dominissini, D. (2017). Epitranscriptomics: Regulation of mRNA metabolism through modifications. Current Opinion in Chemical Biology, 41, 93–98. 10.1016/j.cbpa.2017.10.008 29125941

[wrna1510-bib-0092] Perry, R. P. , & Kelley, D. E. (1975). Methylated constituents of heterogeneous nuclear RNA: Presence in blocked 5′ terminal structures. Cell, 6(1), 13–19. 1164731 10.1016/0092-8674(75)90068-9

[wrna1510-bib-0093] Perry, R. P. , Kelley, D. E. , Friderici, K. , & Rottman, F. (1975). The methylated constituents of L cell messenger RNA: Evidence for an unusual cluster at the 5′ terminus. Cell, 4(4), 387–394. 1168101 10.1016/0092-8674(75)90159-2

[wrna1510-bib-0094] Phalke, S. , Nickel, O. , Walluscheck, D. , Hortig, F. , Onorati, M. , & Reuter, G. (2009). Retrotransposon silencing and telomere integrity in somatic cells of Drosophila depends on the cytosine‐5 methyltransferase DNMT2. Nature Genetics., 41, 696–702. 10.1038/ng.360 19412177

[wrna1510-bib-0095] Polikanov, Y. S. , Melnikov, S. V. , Söll, D. , & Steitz, T. A. (2015). Structural insights into the role of rRNA modifications in protein synthesis and ribosome assembly. Nature Structural & Molecular Biology, 22(4), 342–344. 10.1038/nsmb.2992 PMC4401423 25775268

[wrna1510-bib-0096] Sabban, E. L. , & Bhanot, O. S. (1982). The effect of bisulfite‐induced C to U transitions on aminoacylation of *Escherichia coli* glycine tRNA. The Journal of Biological Chemistry, 257(9), 4796–4805. 7040386

[wrna1510-bib-0097] Sakita‐Suto, S. , Kanda, A. , Suzuki, F. , Sato, S. , Takata, T. , & Tatsuka, M. (2007). Aurora‐B regulates RNA methyltransferase NSUN2. Molecular Biology of the Cell, 18(3), 1107–1117. 10.1091/mbc.e06-11-1021 17215513 PMC1805108

[wrna1510-bib-0098] Salditt‐Georgieff, M. , Jelinek, W. , Darnell, J. E. , Furuichi, Y. , Morgan, M. , & Shatkin, A. (1976). Methyl labeling of HeLa cell hnRNA: A comparison with mRNA. Cell, 7(2), 227–237. 954080 10.1016/0092-8674(76)90022-2

[wrna1510-bib-0099] Saletore, Y. , Meyer, K. , Korlach, J. , Vilfan, I. D. , Jaffrey, S. , & Mason, C. E. (2012). The birth of the Epitranscriptome: Deciphering the function of RNA modifications. Genome Biology, 13(10), 175 10.1186/gb-2012-13-10-175 23113984 PMC3491402

[wrna1510-bib-0100] Sato, G. , Saijo, Y. , Uchiyama, B. , Kumano, N. , Sugawara, S. , Fujimura, S. , … Nukiwa, T. (1999). Prognostic value of nucleolar protein p120 in patients with resected lung adenocarcinoma. Journal of Clinical Oncology: Official Journal of the American Society of Clinical Oncology, 17(9), 2721–2727. 10.1200/JCO.1999.17.9.2721 10561346

[wrna1510-bib-0101] Schaefer, M. (2015). RNA 5‐Methylcytosine analysis by bisulfite sequencing. Methods in Enzymology, 560, 297–329. 10.1016/bs.mie.2015.03.007 26253976

[wrna1510-bib-0102] Schaefer, M. , Pollex, T. , Hanna, K. , & Lyko, F. (2009). RNA cytosine methylation analysis by bisulfite sequencing. Nucleic Acids Research, 37(2), e12 10.1093/nar/gkn954 19059995 PMC2632927

[wrna1510-bib-0103] Schaefer, M. , Pollex, T. , Hanna, K. , Tuorto, F. , Meusburger, M. , Helm, M. , & Lyko, F. (2010). RNA methylation by Dnmt2 protects transfer RNAs against stress‐induced cleavage. Genes and Development, 24(15), 1590–1595. 10.1101/gad.586710 20679393 PMC2912555

[wrna1510-bib-0104] Schosserer, M. , Minois, N. , Angerer, T. B. , Amring, M. , Dellago, H. , Harreither, E. , … Grillari, J. (2015). Methylation of ribosomal RNA by NSUN5 is a conserved mechanism modulating organismal lifespan. Nature Communications, 6, 6158 10.1038/ncomms7158 PMC4317494 25635753

[wrna1510-bib-0105] Schubert, C. (2009). The genomic basis of the Williams‐Beuren syndrome. Cellular and Molecular Life Sciences : CMLS, 66(7), 1178–1197. 10.1007/s00018-008-8401-y 19039520 PMC11131529

[wrna1510-bib-0106] Schwartz, S. (2016). Cracking the epitranscriptome. RNA (New York, NY), 22(2), 169–174. 10.1261/rna.054502.115 PMC4712667 26787305

[wrna1510-bib-0107] Schwartz, S. , Bernstein, D. A. , Mumbach, M. R. , Jovanovic, M. , Herbst, R. H. , León‐Ricardo, B. X. , … Regev, A. (2014). Transcriptome‐wide mapping reveals widespread dynamic‐regulated pseudouridylation of ncRNA and mRNA. Cell, 159(1), 148–162. 10.1016/j.cell.2014.08.028 25219674 PMC4180118

[wrna1510-bib-0108] Shalev‐Benami, M. , Zhang, Y. , Matzov, D. , Halfon, Y. , Zackay, A. , Rozenberg, H. , … Skiniotis, G. (2016). 2.8‐Å Cryo‐EM structure of the large ribosomal subunit from the eukaryotic parasite Leishmania. Cell Reports, 16(2), 288–294. 10.1016/j.celrep.2016.06.014 27373148 PMC5835689

[wrna1510-bib-0109] Shanmugam, R. , Fierer, J. , Kaiser, S. , Helm, M. , Jurkowski, T. P. , & Jeltsch, A. (2015). Cytosine methylation of tRNA‐asp by DNMT2 has a role in translation of proteins containing poly‐asp sequences. Cell Discovery, 1(1), 15010 10.1038/celldisc.2015.10 27462411 PMC4860778

[wrna1510-bib-0110] Sharma, S. , & Lafontaine, D. L. J. (2015). “View from a bridge”: A new perspective on eukaryotic rRNA base modification. Trends in Biochemical Sciences, 40(10), 560–575. 10.1016/j.tibs.2015.07.008 26410597

[wrna1510-bib-0111] Sharma, S. , Yang, J. , Watzinger, P. , Kötter, P. , & Entian, K.‐D. (2013). Yeast Nop2 and Rcm1 methylate C2870 and C2278 of the 25S rRNA, respectively. Nucleic Acids Research, 41(19), 9062–9076. 10.1093/nar/gkt679 23913415 PMC3799443

[wrna1510-bib-0112] Shima, J. E. , McLean, D. J. , McCarrey, J. R. , & Griswold, M. D. (2004). The murine testicular transcriptome: Characterizing gene expression in the testis during the progression of spermatogenesis. Biology of Reproduction, 71(1), 319–330. 10.1095/biolreprod.103.026880 15028632

[wrna1510-bib-0113] Sloan, K. E. , Warda, A. S. , Sharma, S. , Entian, K.‐D. , Lafontaine, D. L. J. , & Bohnsack, M. T. (2017). Tuning the ribosome: The influence of rRNA modification on eukaryotic ribosome biogenesis and function. RNA Biology, 14(9), 1138–1152. 10.1080/15476286.2016.1259781 27911188 PMC5699541

[wrna1510-bib-0114] Sobala, A. , & Hutvagner, G. (2013). Small RNAs derived from the 5′ end of tRNA can inhibit protein translation in human cells. RNA Biology, 10(4), 553–563. 10.4161/rna.24285 23563448 PMC3710361

[wrna1510-bib-0115] Sokołowski, M. , Klassen, R. , Bruch, A. , Schaffrath, R. , & Glatt, S. (2018). Cooperativity between different tRNA modifications and their modification pathways. BBA—Gene Regulatory Mechanisms, 1861(4), 409–418. 10.1016/j.bbagrm.2017.12.003 29222069

[wrna1510-bib-0116] Song, J. , & Yi, C. (2017). Chemical modifications to RNA: A new layer of gene expression regulation. ACS Chemical Biology, 12(2), 316–325. 10.1021/acschembio.6b00960 28051309

[wrna1510-bib-0117] Squires, J. E. , Patel, H. R. , Nousch, M. , Sibbritt, T. , Humphreys, D. T. , Parker, B. J. , … Preiss, T. (2012). Widespread occurrence of 5‐methylcytosine in human coding and non‐coding RNA. Nucleic Acids Research, 40(11), 5023–5033. 10.1093/nar/gks144 22344696 PMC3367185

[wrna1510-bib-0118] Suzuki, T. , Nagao, A. , & Suzuki, T. (2011). Human mitochondrial tRNAs: Biogenesis, function, structural aspectsand diseases. Annual Review of Genetics, 45(1), 299–329. 10.1146/annurev-genet-110410-132531 21910628

[wrna1510-bib-0119] Takemoto, C. , Spremulli, L. L. , Benkowski, L. A. , Ueda, T. , Yokogawa, T. , & Watanabe, K. (2009). Unconventional decoding of the AUA codon as methionine by mitochondrial tRNAMet with the anticodon f5CAU as revealed with a mitochondrial in vitro translation system. Nucleic Acids Research, 37(5), 1616–1627. 10.1093/nar/gkp001 19151083 PMC2655697

[wrna1510-bib-0120] Tang, H. , Fan, X. , Xing, J. , Liu, Z. , Jiang, B. , Dou, Y. , … Wang, W. (2015). NSun2 delays replicative senescence by repressing p27 (KIP1) translation and elevating CDK1 translation. Aging, 7(12), 1143–1158. 10.18632/aging.100860 26687548 PMC4712338

[wrna1510-bib-0121] Traube, F. R. , & Carell, T. (2017). The chemistries and consequences of DNA and RNA methylation and demethylation. RNA Biology, 14(9), 1099–1107. 10.1080/15476286.2017.1318241 28440690 PMC5699545

[wrna1510-bib-0122] Trixl, L. , Amort, T. , Wille, A. , Zinni, M. , Ebner, S. , Hechenberger, C. , … Lusser, A. (2018). RNA cytosine methyltransferase Nsun3 regulates embryonic stem cell differentiation by promoting mitochondrial activity. Cellular and Molecular Life Sciences: CMLS, 75(8), 1483–1497. 10.1007/s00018-017-2700-0 29103146 PMC5852174

[wrna1510-bib-0123] Tuorto, F. , Herbst, F. , Alerasool, N. , Bender, S. , Popp, O. , Federico, G. , … Lyko, F. (2015). The tRNA methyltransferase Dnmt2 is required for accurate polypeptide synthesis during haematopoiesis. The EMBO Journal, 34(18), 2350–2362. 10.15252/embj.201591382 26271101 PMC4570521

[wrna1510-bib-0124] Tuorto, F. , Liebers, R. , Musch, T. , Schaefer, M. , Hofmann, S. , Kellner, S. , … Lyko, F. (2012). RNA cytosine methylation by Dnmt2 and NSun2 promotes tRNA stability and protein synthesis. Nature Structural & Molecular Biology, 19(9), 900–905. 10.1038/nsmb.2357 22885326

[wrna1510-bib-0125] Uchiyama, B. , Saijo, Y. , Kumano, N. , Abe, T. , Fujimura, S. , Ohkuda, K. , … Nukiwa, T. (1997). Expression of nucleolar protein p120 in human lung cancer: Difference in histological types as a marker for proliferation. Clinical Cancer Research: An Official Journal of the American Association for Cancer Research, 3(10), 1873–1877. 9815576

[wrna1510-bib-0126] Ueki, T. , Nakayama, Y. , Sugao, Y. , Kohno, K. , Matsuo, K. , Sugimoto, Y. , … Tsuneyoshi, M. (1997). Significance of the expression of proliferation‐associated nucleolar antigen p120 in human colorectal tumors. Human Pathology, 28(1), 74–79. 9013835 10.1016/s0046-8177(97)90282-3

[wrna1510-bib-0127] Van Haute, L. , Dietmann, S. , Kremer, L. , Hussain, S. , Pearce, S. F. , Powell, C. A. , … Minczuk, M. (2016). Deficient methylation and formylation of mt‐tRNA(met) wobble cytosine in a patient carrying mutations in NSUN3. Nature Communications, 7, 12039 10.1038/ncomms12039 PMC4931328 27356879

[wrna1510-bib-0128] Van Haute, L. , Powell, C. A. , & Minczuk, M. (2017). Dealing with an unconventional genetic code in mitochondria: The biogenesis and pathogenic defects of the 5‐Formylcytosine modification in mitochondrial tRNAMet. Biomolecules, 7(1), 24 10.3390/biom7010024

[wrna1510-bib-0129] Väre, V. Y. P. , Eruysal, E. R. , Narendran, A. , Sarachan, K. L. , & Agris, P. F. (2017). Chemical and conformational diversity of modified nucleosides affects tRNA structure and function. Biomolecules, 7(1), 29 10.3390/biom7010029 PMC5372741 28300792

[wrna1510-bib-0130] Wang, N. , Tang, H. , Wang, X. , Wang, W. , & Feng, J. (2017). Homocysteine upregulates interleukin‐17A expression via NSun2‐mediated RNA methylation in T lymphocytes. Biochemical and Biophysical Research Communications, 493(1), 94–99. 10.1016/j.bbrc.2017.09.069 28919411

[wrna1510-bib-0131] Wang, S. , & Kool, E. T. (1995). Origins of the large differences in stability of DNA and RNA helices: C‐5 methyl and 2′‐hydroxyl effects. Biochemistry, 34(12), 4125–4132. 7535100 10.1021/bi00012a031

[wrna1510-bib-0132] Weber, M. , Davies, J. J. , Wittig, D. , Oakeley, E. J. , Haase, M. , Lam, W. L. , & Schübeler, D. (2005). Chromosome‐wide and promoter‐specific analyses identify sites of differential DNA methylation in normal and transformed human cells. Nature Genetics, 37(8), 853–862. 10.1038/ng1598 16007088

[wrna1510-bib-0133] Wetzel, C. , & Limbach, P. A. (2013). The global identification of tRNA isoacceptors by targeted tandem mass spectrometry. The Analyst, 138(20), 6063–6072. 10.1039/c3an01224g 23954863

[wrna1510-bib-0134] Wilkinson, C. R. , Bartlett, R. , Nurse, P. , & Bird, A. P. (1995). The fission yeast gene pmt1+ encodes a DNA methyltransferase homologue. Nucleic Acids Research, 23(2), 203–210. 7862522 10.1093/nar/23.2.203 PMC306655

[wrna1510-bib-0135] Wu, P. , Brockenbrough, J. S. , Paddy, M. R. , & Aris, J. P. (1998). NCL1, a novel gene for a non‐essential nuclear protein in *Saccharomyces cerevisiae* . Gene, 220(1–2), 109–117. 9767141 10.1016/s0378-1119(98)00330-8

[wrna1510-bib-0136] Wyatt, G. R. (1950). Occurrence of 5‐methylcytosine in nucleic acids. Nature, 166(4214), 237–238. 10.1038/166237b0 15439258

[wrna1510-bib-0137] Xing, J. , Yi, J. , Cai, X. , Tang, H. , Liu, Z. , Zhang, X. , … Wang, W. (2015). NSun2 promotes cell growth via elevating cyclin‐dependent kinase 1 translation. Molecular and Cellular Biology, 35(23), 4043–4052. 10.1128/MCB.00742-15 26391950 PMC4628067

[wrna1510-bib-0138] Xiong, X. , Li, X. , & Yi, C. (2018). N1‐methyladenosine methylome in messenger RNA and non‐coding RNA. Current Opinion in Chemical Biology, 45, 179–186. 10.1016/j.cbpa.2018.06.017 30007213

[wrna1510-bib-0139] Yang, X. , Yang, Y. , Sun, B.‐F. , Chen, Y.‐S. , Xu, J.‐W. , Lai, W.‐Y. , … Yang, Y. G. (2017). 5‐methylcytosine promotes mRNA export—NSUN2 as the methyltransferase and ALYREF as an m(5)C reader. Cell Research, 27(5), 606–625. 10.1038/cr.2017.55 28418038 PMC5594206

[wrna1510-bib-0140] Zachau, H. G. , Dütting, D. , & Feldmann, H. (1966). Nucleotidsequenzen zweier serinspezifischer Transfer‐Ribonucleinsäuren. Angewandte Chemie, 78(7), 392–393. 10.1002/ange.19660780714

[wrna1510-bib-0141] Zhao, B. S. , Roundtree, I. A. , & He, C. (2016). Post‐transcriptional gene regulation by mRNA modifications. Nature Reviews Molecular Cell Biology, 18(1), 31–42. 10.1038/nrm.2016.132 27808276 PMC5167638

